# The Causal Mechanism Between the Dipeptidyl Peptidase-4, Heart Failure, and Other Cardiovascular Diseases: A Mendelian Randomization and Mediation Study

**DOI:** 10.1155/ije/2357272

**Published:** 2025-08-26

**Authors:** Che-Kai Chen, Chang-Fu Kuo, Yu-Jing Chang, Weiya Zhang, Michael Doherty, Ming-Ling Chang, Tsung-Hsing Chen

**Affiliations:** ^1^Center for Artificial Intelligence in Medicine, Linkou Chang Gung Memorial Hospital, Taoyuan, Taiwan; ^2^School of Medicine, Chang Gung University, Taoyuan, Taiwan; ^3^Academic Rheumatology, School of Medicine, University of Nottingham, Nottingham, UK; ^4^Pain Centre Versus Arthritis, University of Nottingham, Nottingham, UK; ^5^Department of Gastroenterology and Hepatology, Linkou Chang Gung Memorial Hospital, Chang Gung University College of Medicine, Taoyuan, Taiwan

**Keywords:** cardiovascular diseases (CVDs), dipeptidyl peptidase-4 (DPP4) inhibitors, mediation analysis, Mendelian randomization (MR), type-2 diabetes

## Abstract

**Aims:** Dipeptidyl peptidase-4 (DPP4) inhibitors are commonly used to treat type 2 diabetes. However, the causality of it on cardiovascular diseases (CVDs) is controversial. This study aimed (1) to investigate the causal mechanisms of DPP4 gene expression at the mRNA level on CVDs, including all-cause heart failure (HF), atrial fibrillation (AF), myocardial infarction (MI), and stroke in a European population; (2) to assess the direct effect of DPP4 at the mRNA level on CVD, which is independent of type-2 diabetes; and (3) to explore the causality of DPP4 inhibition on CVDs and type-2 diabetes.

**Methods:** Utilizing DPP4 and CVD summary statistics from eQTLGen Consortium, GTEx Portal, and UK Biobank, we applied weak IV and pleiotropy robust Mendelian randomization methods (MR-RAPS, GRAPPLE, BESIDE-MR, debiased IVW) and mediation analysis to assess the causal impact of DPP4 at the mRNA level on CVD and the direct effect of DPP4 at the mRNA level on CVD, not mediated by diabetes. The causality of DPP4 inhibition on CVD was also evaluated.

**Results:** MR-RAPS suggested a potential causal relationship between increased DPP4 at the mRNA levels and HF (0.031 [95% CI, 0.06–0.56; *p*=0.014]). However, there was limited evidence that increased DPP4 levels affect AF, MI, or stroke. Other analyses corroborated these findings. Mediation analysis indicated a direct effect of DPP4 at the mRNA level on HF, while debiased IVW showed limited evidence for a causal effect of DPP4 inhibition on CVDs, possibly due to low statistical power.

**Conclusions:** Mendelian randomization analyses support the cardiovascular safety of DPP4 inhibitors in managing type 2 diabetes, with little evidence for DPP4-mediated cardiovascular harm, reinforcing their appropriateness for clinical use in European populations. Additionally, if DPP4 inhibition affects cardiovascular outcomes, it may not do so through glycemic control, such as HbA1c reduction.

**Summary**

• What is already known?
◦ DPP4 inhibitors are commonly used to treat type 2 diabetes. However, the causal association needs to be explored further.
• What this study has found?
◦ Mendelian randomization study found that increased DPP4 at the mRNA level is causally linked to heart failure but not to atrial fibrillation, myocardial infarction, or stroke in Europeans.
◦ Mediation analysis revealed that the DPP4 at the mRNA level directly affects HF, and this relationship is not mediated by type 2 diabetes mellitus.
◦ DPP4 inhibition is not causally linked to cardiovascular diseases.
• What are the implications of the study?
◦ DPP4 inhibitors remain a safe option for managing blood sugar in type 2 diabetes.
◦ Minimal risk of DPP4-mediated CVDs, supporting their suitability for diabetes treatment.

## 1. Introduction

Type 2 diabetes mellitus is a significant health concern, with its patients being at a heightened risk for cardiovascular diseases (CVDs), including heart failure (HF), as a notably adverse outcome [1, 2]. The enzyme dipeptidyl peptidase-4 (DPP4) is pivotal in this context. DPP4 regulates blood glucose levels by modulating the activity of hormones like glucagon-like peptide (GLP)-1 and gastric inhibitory polypeptide (GIP). These hormones stimulate insulin release and decrease glucagon secretion, ensuring balanced glucose levels. Moreover, DPP4 has been recognized as a potential contributor to CVD through a series of stress-induced reactions.

Given the significance of DPP4 in glucose regulation, DPP-4 inhibitors have emerged as promising therapeutic agents for treating type-2 diabetes [3, 4]. These inhibitors target the DPP-4 enzyme, successfully lowering blood sugar levels, and have shown improvements in glycated hemoglobin levels in clinical trials without exacerbating hypoglycemia or contributing to weight gain. However, the influence of DPP-4 inhibitors on cardiovascular health has been a topic of debate and research [5]. Preliminary findings suggest potential cardiovascular benefits, possibly due to improved metabolic parameters, such as reduced weight gain and decreased inflammation. While the safety profile of DPP4 inhibitors predominantly aligns with clinical expectations, the SAVOR-TIMI 53 trial reported that saxagliptin is associated with an increased hazard ratio (1.27) for HF-related hospitalization [6–8]. However, subsequent clinical trials reported inconclusive results on the association between DPP4 inhibitors and the risk of hospitalization for HF [9, 10]. Observational studies presented ambiguous associations between DPP4 inhibitors and HF hospitalization risk [11, 12]. Moreover, two meta-analyses presented varied conclusions on the correlation between DPP4 inhibitors and CVD manifestation [13, 14]. In Japan, diabetic patients who use DPP-4 inhibitors may have a lower risk of experiencing their first cardiovascular events [15, 16]. In recent years, other glucose-lowering drug classes have also been recognized for their cardiovascular benefits. In particular, SGLT2 inhibitors and GLP-1 receptor agonists have demonstrated significant cardiovascular effects in several clinical trials, including marked reductions in HF hospitalizations. This emerging evidence offers a broader context for interpreting the cardiovascular impact of DPP4 inhibitors and highlights the need to consider the mechanistic differences among various therapeutic approaches [17, 18]. This study seeks to delve deeper into these issues; we utilize a series of weak IV and pleiotropy robust Mendelian randomization (MR) methods and causal mediation analysis by MR approach to explore the causal mechanisms (causal relationship and biological mechanisms) of DPP4 and various CVDs, including HF, atrial fibrillation (AF), myocardial infarction (MI), and stroke within a European population.

## 2. Materials and Methods

### 2.1. Study Design and Participants

We addressed clinically pertinent queries framed in a causal context: (1) Does the level of DPP4 gene expression at the mRNA level exert a causal influence on the development of CVD? (2) Is the impact of the DPP4 gene expression at the mRNA level on CVD independent of the mediation by type-2 diabetes? (3) Is there a causal association between DPP4 inhibition and the onset of CVDs? To elucidate the first query, we employed two-sample MR to investigate the potential causal mechanism between DPP4 gene expression at the mRNA level and CVD. For the distinct mRNA level of DPP4 gene expression, the expression quantitative trait locus (eQTL) summary statistics for the first exposure DPP4 gene expression at the mRNA level were collected from the eQTLGen Consortium, which included 31,684 individuals of European descent [17].

For the second query, MR-based mediation analysis was used to discern the direct influence of DPP4 gene expression at the mRNA level on CVD without the intermediary effect of type-2 diabetes. This study's schematic representation is provided in Figure S1 in Supporting 1. Furthering our investigation into the role of DPP4 inhibition (third query), we align with methodologies outlined in studies by Walker et al. [18] and Xu et al. [19]. We define a novel exposure, “DPP4 inhibition,” associating it with relevant single-nucleotide polymorphisms (SNPs) to ensure inverse correlations between each SNP's effect on HbA1c and the mediation by the DPP4 gene, to proxy the effect of DPP4 inhibitors. We collected specific SNP in each tissue for the DPP4 gene with gene ID ENSG00000197635.9 from the Genotype-Tissue Expression (GTEx) Portal (files: GTEx_Analysis_v8_eQTL) [20]. Furthermore, we used data for HbA1c collected from the MRC Integrative Epidemiology Unit (IEU) OpenGWAS project [21] to define DPP4 inhibition. The debiased inverse variance weight method (debiased IVW) is then applied to discern the causal relationship between DPP4 inhibition, CVDs, and type-2 diabetes.

The schematic representation of the overall study is provided in Figure S2 of Supporting 1. Detailed methodologies are presented in Method S1, Supporting 1. When reporting our findings, we followed the Strengthening the Reporting of Observational Studies in Epidemiology guidelines for MR (STROBE-MR, Table S1 in Supporting 1) [22].

### 2.2. Phenotyping

For outcome variables—all-cause HF, AF, MI, and stroke—we accessed genome-wide association study (GWAS) summary statistics from the UK Biobank (UKB) dataset, which included data from 456,005 UK participants (Method S2, Supporting 1). Type-2 diabetes data were sourced both from the individual UKB dataset and the FinnGen project (FinnGen) R9 [23]. A stringent quality control (QC) protocol was applied to the UKB individual data, encompassing 443,107 participants.  Table S2 in Supporting 1 provides information on the GWAS summary datasets used in the study. The case definitions of CVD in this study are based on Aragam et al. [24]. The case definitions of type-2 diabetes from UKB and FinnGen R9 were based on the International Classification of Diseases 10th revisions (ICD-10) codes E11 and E14 (Table S3 in Supporting 1). Disease phenotyping in both data sources was based on linkage to clinical records.

### 2.3. CVD and Type-2 Diabetes GWAS From the Individual Datasets From the UKB

GWAS and a standard QC procedure to identify SNPs of interest for all-cause HF, AF, MI, and stroke were performed using PLINK 2.0. [25]. The SNPs were evaluated according to the Genotyping Call Rate (GCR), Hardy–Weinberg Equilibrium (HWE), and the Minor Allele Frequency (MAF). SNPs were excluded if the following conditions were met: GCR < 0.95; HWE < 10^−6^; MAF < 0.01; or SNPs allocated on sex chromosomes. Following the QC procedure, 6,215,253 imputed SNPs and 443,107 UKB participants were included in the analysis. The genotype derived from each SNP was treated as a nominal variable (i.e., 0, 1, or 2). Logistic regression was performed to determine the association between CVD and SNPs and the association between type-2 diabetes and SNPs, and all regressions were adjusted for age, sex, body mass index (BMI), and the top 10 principal components (PCs) from PC analysis to reduce bias from population stratification [26].

### 2.4. MR Analysis

MR utilizes genetic variants (commonly SNPs) as instrumental variables (IVs) to infer causal relationships between exposures and outcomes [27–29]. MR capitalizes on the random distribution of genetic variants within populations to counteract bias from unaccounted confounding. Conventional MR relies on three assumptions: genetic variants' association with the exposure, their independence from unmeasured confounders, and their exclusive effect on outcomes only through exposure. Traditional MR techniques may face challenges like weak IV bias, pleiotropy effects, the Instrument Strength Independent of Direct Effect (InSIDE) assumption, and the winner's curse. In particular, restricting IVs to only genome-wide significant SNPs may exacerbate selection bias. These concerns have been systematically addressed in recent methodological developments, such as Mendelian randomization with the Robust Adjusted Profile Score (MR-RAPS) [30]. To address these, we employed a range of weak IVs and pleiotropy robust MR methods, including MR-RAPS, the framework Genome-wide mR Analysis under Pervasive PLEiotropy (GRAPPLE) [31], Bayesian set identification Mendelian randomization (BESIDE-MR) [32], and debiased IVW [29, 33]. The specifics are detailed in Methods S3 and S4 in Supporting 1. The primary analysis used MR-RAPS, employing Tukey's Biweight loss for robustness against outliers, with other techniques serving supporting roles. Both GRAPPLE and BESIDE-MR are expansive MR-RAPS frameworks, while BESIDE-MR additionally discerns the number of SNP groups to integrate. The debiased IVW estimator, an IVW variant, reduces the winner's curse and pleiotropy biases and is resilient to weak instruments. The GRAPPLE workflow was utilized to detect pleiotropic pathways. If a singular mode appeared, the causal effect was directly estimated. In multimodal cases, potential confounding risk factors were incorporated for accurate causal estimations. The modified conditional Cochran's *Q* statistic for multi-MR was evaluated to assess the strength of IVs by detecting heterogeneity [34, 35]. In addition to identifying multiple pleiotropic pathways, the GRAPPLE framework also allows inference on the direction of causality by examining the shape of the robust profile likelihood. Under the correct causal direction, the profile typically exhibits a single mode near the actual causal effect. In contrast, an incorrect specification often produces a bimodal distribution with one peak near zero [31].

The BESIDE-MR used both one and two components. The posterior probability of inclusion (PPI) of an SNP gauged the likelihood of its heterogeneity, guiding SNP classification. We also used BESIDE-MR with penalization as an additional analysis to explore the sensitivity of the BMA to the average number of SNPs included in the model [32].  Method S3 in Supporting 1 illustrates the overall structure of RAPS-based MR methods. Considering the number of available IVs and the assumptions of the MR model, we employed RAPS-based methods to investigate the causal role of DPP4 gene expression at the mRNA level in CVD risk. Specifically, MR-RAPS was used as the primary analysis, with GRAPPLE and BESIDE-MR conducted as sensitivity analyses. To enhance the robustness of our findings, we also performed additional analyses using debiased IVW, which incorporates both weak and strong IVs, and conventional MR methods, such as classical IVW based on genome-wide significant SNPs. Furthermore, debiased IVW was used to assess the potential causal effect of DPP4 inhibition on CVD risk. Triangulation in etiological epidemiology is considered in this study. Consistency in the results from these methods increases confidence in assessing causality [36, 37]. Potential risk factors from SNP heterogeneity were extracted using PhenoScanner V2 and the disgenet2r package. The causal effect for binary outcomes was determined using the mean effect/beta, with the causal odds ratio (OR) stemming from its exponential function.

### 2.5. Causal Mediation Analysis by MR Approach

Causal mediation analysis elucidates the mechanisms linking an exposure to an outcome, both directly and through a mediator. This analysis quantifies three key effects: total, direct, and indirect (or path-specific for multi-mediators) [38]. Our study designated the DPP4 gene expression at the mRNA level as the exposure, type-2 diabetes as the mediator, and CVDs as the outcomes (Method S5 in Supporting 1). We aimed to determine the direct influence of the DPP4 gene expression at the mRNA level on each CVD bypassing the mediation of type-2 diabetes. Here, we implemented a multivariate MR strategy within the difference-in-coefficients method framework for this mediation analysis (causal mediation analysis by MR approach) [39] to estimate the direct effect of DPP4 gene expression at the mRNA level on each CVD (Method S5 in Supporting 1). Notably, the identification assumptions for MR-based mediation analysis differ from those of conventional mediation analysis, which requires strong assumptions about the absence of unmeasured confounding. In contrast, causal mediation analysis using the MR approach can yield less biased causal estimates even when such confounding exists between the exposure, mediator, and outcome. We performed multivariable MR (multi-GRAPPLE) to estimate the direct effect of DPP4 gene expression on CVD risk, adjusting for the genetic effects of type 2 diabetes using the difference-in-coefficients method. The direct effect was the estimate of DPP4 gene expression on the risk of each CVD with adjustment for type-2 diabetes. The direct effect was then subtracted from the total effect, estimated using MR, to determine the indirect effect of DPP4 gene expression on the risk of each CVD (Method S5 in Supporting 1).

### 2.6. SNP Selection for DPP4 Gene Expression at the mRNA Level

From the eQTLGen Consortium, we sourced about 15,000 eQTL summaries for the DPP4 gene expression at the mRNA level. Although RAPS-based MR methods are designed to accommodate weak IVs, we still applied a relatively stringent *p* value threshold of 10^−4^ for SNP selection. This criterion was necessary to ensure a balance between including a sufficient number of IVs and maintaining adequate IV strength. There are two primary reasons for this requirement [30, 31, 40]. The first reason is to increase power, as including too many weak SNPs would increase the variance of the causal estimate. The second reason is that we do not want other, unknown exposures to introduce large pleiotropic effects with SNPs that are unassociated or weakly associated with the target exposure. To address both concerns, we adopted a moderate *p* value threshold, such as 10^−4^, which increases the number of available IVs while preserving their relevance to the exposure. Additionally, using a moderate *p* value threshold may substantially increase the number of available SNPs, which could be advantageous for identifying potential biological pathways. Due to convergence issues observed in some two-sample MR models, different *p* value thresholds were applied depending on the specific analysis. We also took measures to sidestep linkage disequilibrium (LD) by favoring SNPs with an *R*^2^ < 0.001 and harmonizing the alleles and effects between the exposure and outcome. For multi-GRAPPLE analysis with multiple risk factors, Bonferroni correction was applied [31]. For comprehensive SNP details and their precise selection criteria, refer to Table S4 in Supporting 1 and Tables S1–S4 in Supporting 2, in which two SNPs, rs12619850 and rs35505926, are genome-wide significant (*p* value < 5 × 10^−8^).

### 2.7. SNP Selection for DPP4 Inhibition

To define DPP4 inhibition, our initial step was to gather the leading SNPs associated with the DPP4 gene from the GTEx dataset. We focused on SNPs showcasing a negative effect on DPP4's inhibitory role via HbA1c. SNPs with weak LD, gauged by a squared correlation coefficient *R*^2^ < 0.001, were preferred. Given the limited number of available SNPs and the robustness of our method against weak IVs, we did not impose any *p* value filtering for this process. Our efforts culminated in identifying two suitable SNPs for use as IVs containing a weak IV. Details are available in Part I of Method S1 in Supporting 1.

### 2.8. Assessment of MR Assumptions

The validity of MR assumptions was assessed using MR-RAPS, emphasizing the InSIDE assumption through diagnostic and quantile-quantile plots [40] (Method S6 in Supporting 1). For relevance assumption, all methodologies employed were rooted in MR-RAPS, showing enhanced resilience to weak IVs compared to traditional MR. Exchangeability assumption was addressed by adjusting for age, sex, BMI, and the top 10 PCs during the GWAS procedure for UKB participants, thus curbing the influence of population stratification and potential SNP–outcome confounding relationships. For exclusion restriction, sensitivity analyses aimed to mitigate the bias from uncorrelated horizontal pleiotropy and delve into potential pathways. The correlated horizontal pleiotropy bias was tackled with GRAPPLE by adjusting potential phenotypes as confounders. Furthermore, BESIDE-MR served to minimize bias impact, discussing the instrument validity of SNPs in the PPI context. All MR and GWAS procedures were executed using R (version 4.0.3) and PLINK 2.0 [25].

## 3. Results

### 3.1. Participant Characteristics

Participant characteristics from the UKB datasets are detailed in Table S5 in Supporting 1. The dataset includes 443,107 participants, with 6,215,253 identified genetic variants for CVD. The average age was 57.9 (standard deviation [SD]: 8.4); 54% were women. The average BMI was 27.4 kg/m^2^ (SD 4.8). Prevalences of HF, AF, MI, and stroke were 3.5%, 7.5%, 5.4%, and 3.7%, respectively. Type-2 diabetes prevalence was 7% in UKB and 12% in FinnGen. Characteristics of eQTLGen, UKB, and FinnGen datasets can be found in Method S2 in Supporting 1.

### 3.2. Identifying Potential Pleiotropic Pathways for the Effect of DPP4 Gene Expression at the mRNA Level on CVD

**Figure 1:**
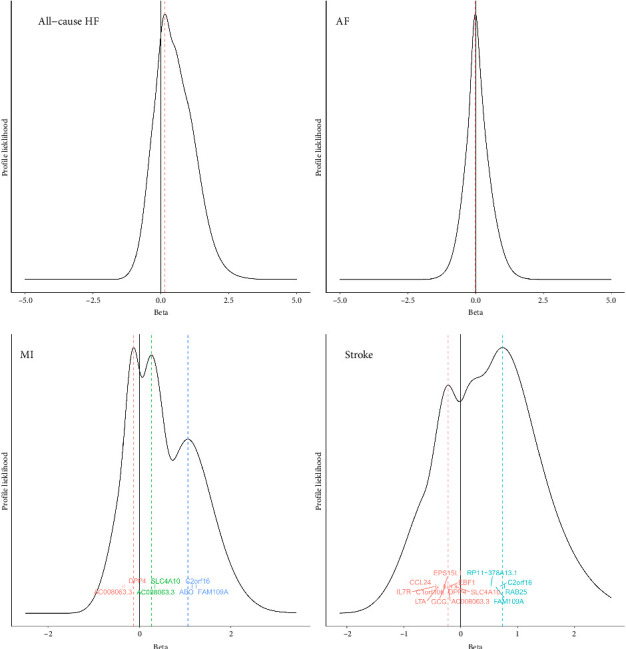
GRAPPLE mode-detection plot, including a profile-likelihood graph and the modes of profile likelihood for the effect of DPP4 gene expression at the mRNA level on CVD. The *x*-axis and *y*-axis represent the effect/beta and the value of the profile-likelihood function, respectively. The modes that occur at points (the intersections of the dotted lines with the beta-axis) are represented by the intersections of the dotted lines of different colors with the graph, and the different colored words indicate marker genes (Tables S5–S9 in Supporting 2) related to the different modes.

The GRAPPLE identified potential pleiotropic pathways for disease triggered by heterogeneous genetic instruments. No pleiotropic pathway exists from DPP4 gene expression at the mRNA level to all-cause HF or AF, as per the GRAPPLE mode plots (Figure 1). The profile likelihoods for both outcomes exhibited a single, prominent mode without secondary peaks near zero. This unimodal pattern supports the assumed causal direction from DPP4 expression to HF and AF, reducing the likelihood of reverse causality in these relationships. However, evaluations revealed potential pathways between DPP4 gene expression at the mRNA level and MI, as well as DPP4 gene expression at the mRNA level and stroke. Specific genes, SNPs, and diseases are detailed in Tables S5–S9 in Supporting 2. GRAPPLE suggests pathways from DPP4 gene expression at the mRNA level to MI and stroke are notably influenced by factors such as BMI, SLC4A10, total cholesterol (TC), hypertension (HTN), and total triglyceride (TG). Also, the one-component BESIDE-MR produced similar results to GRAPPLE for the pathway from DPP4 gene expression at the mRNA level to MI and stroke. All details are provided in Method S7 in Supporting 1 and in Tables S5–S7 and S10–S19 in Supporting 2.

### 3.3. Assess the Causality of DPP4 Gene Expression at the mRNA Level on CVDs

#### 3.3.1. The Causal Effect of DPP4 Gene Expression at the mRNA Level on All-Cause HF and Other CVD

**Figure 2:**
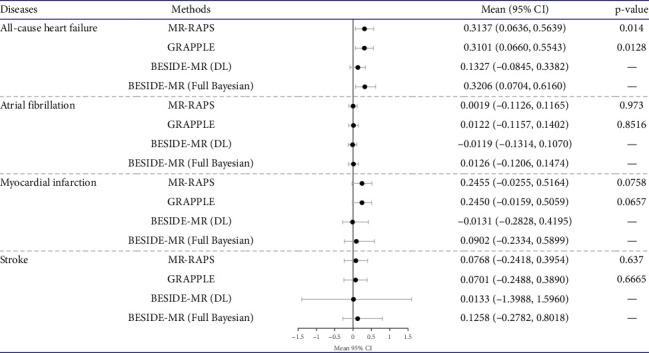
The causal effect of DPP4 gene expression at the mRNA level on CVDs, including HF, AF, MI, and stroke via univariate MR. The heterogeneity of the SNPs may have biased results based on GRAPPLE for DPP4 gene expression at the mRNA level on MI or stroke. The mean effects, 95% confidence or credible intervals, and *p* values are listed. Abbreviation: CI: confidence intervals for MR-GENIUS, MR-RAPS, and GRAPPLE; credible interval for BESIDE-MR. Mean: the mean causal effect; *p* value: *p* value of the mean causal effect.

All results based on MR-RAPS as a primary analysis and GRAPPLE and BESIDE-MR (full-Bayesian) as sensitivity analyses showed that increased mRNA levels of DPP4 gene expression increased the risk of all-cause HF in a European population (the effect in Figure 2 and the OR in Figure S3 in Supporting 1). The results based on MR-RAPS showed that DPP4 gene expression at the mRNA level increased with the risk of all-cause HF, with a causal OR of 1.37 (95% confidence interval [CI], 1.07–1.76) (effect/beta, 0.03; 95% CI, 0.06–0.56). Findings leveraging MR-RAPS, GRAPPLE, and BESIDE-MR highlight a potential increase in HF risk with elevated DPP4 levels in European populations. However, the evidence was inconclusive regarding relationships between DPP4 levels and other CVDs (AF, MI, and stroke), despite the potential pleiotropic pathway from DPP4 levels to MI and stroke. Multivariate MR within the GRAPPLE framework revealed the impact of specific candidates like SLC4A10, BMI, TC, HTN, and TG on the MI and stroke pathways from DPP4 gene expression at the mRNA level. The results suggested that while some factors might mediate effects, others, like SLC4A10 and BMI, may not necessarily influence the impact of DPP4 gene expression at the mRNA level on MI (see Method S8 in Supporting 1 and Tables S10 and S11 in Supporting 2 for the main analysis, and Tables S20 and S21 in Supporting 2 for the reliability analysis).

### 3.4. Causal Mediation Analysis

We used two sources for mediator type-2 diabetes to robust our results for the mediation analysis (Method S5 in Supporting 1). By using around 10^−4^ as a criterion to select SNPs as IVs (Tables S22–S25 in Supporting 2), muti-GRAPPLE shows that DPP4 gene expression at the mRNA level affects HF independent to type-2 diabetes (effect: 0.25; CI, 0.01–0.45; *p*=0.04 for UKB. effect: 0.23; CI, 0.02–0.44; *p*=0.03 for FinnGen), which implies the mediation analysis support that DPP4 gene expression at the mRNA level has a direct effect on HF, which is not mediated by type-2 diabetes for both two data sources. However, the evidence remains inconclusive for other conditions like AF, MI, and stroke.

### 3.5. The Causal Estimates of DPP4 Gene Expression at the mRNA Level on HbA1c

We used a series of MR methods to investigate the causality of DPP4 gene expression at the mRNA level on HbA1c. Except for debiased IVW, all RAPS-based MR shows that DPP4 gene expression at the mRNA level positively affects HbA1c (Table 1, Table S26 in Supporting 2 for IVs). Therefore, we choose HbA1c as the class effect of DPP4 inhibition.

### 3.6. Assess the Causality of DPP4 Inhibition on CVDs

We performed debiased IVW to estimate the effect of DPP4 inhibition on CVDs. The results based on GTEx (Table 2) show that DPP4 inhibition was not causal to the risk of all-cause HF (OR: 0.45; CI, 0.15–1.31), AF (OR: 1.97; CI, 0.87–4.51), MI (OR: 2.89; CI: 0.07–127.46), and stroke (OR: 0.78; CI, 0.03–20.70). The results based on eQTLGen show similar results (subsection B in Method S1, Supporting 1).

We adopted two approaches, examining DPP4 gene expression at the mRNA level and DPP4 inhibition concerning CVDs. Varying penalization parameters and employing BESIDE-MR yielded consistent results. We could not conduct two-component BESIDE-MR due to limited SNPs. To address data source limitations, we used debiased IVW. While MR results suggested that increased DPP4 gene expression at the mRNA level may raise the risk of all-cause HF in the European population, causality for other CVDs was inconclusive. Finally, we used debiased IVW to assess the impact of DPP4 inhibition with two IVs containing a weak IV to mitigate potential biases.

### 3.7. Reliability

We adopt two approaches by considering two exposures with related DPP4 (DPP4 gene expression at the mRNA level and DPP4 inhibition) to assess the causal mechanism between DPP4 and CVDs. For model complexity and to strengthen our results from BESIDE-MR, the one-component BESIDE-MR with penalization term (penalization parameters between −3 and 3) also showed consistency with the previous result for each case (Tables S16–S19 in Supporting 2). However, we could not conduct a two-component BESIDE-MR because the number of SNPs was insufficient. Although we have no additional GWAS to identify independent IVs in a three-sample design [40], we used debiased IVW to address the limitation on data sources to reduce the winner's curse bias. Since the number of significant SNPs was less than 3, we only reported the classical IVW using the significant SNP and debiased IVW in Tables S20 and S21 in Supporting 2, and the results were consistent.

## 4. Discussion

This study examined the causal relationship between DPP4 gene expression at the mRNA level, its inhibition, and the risk of CVDs using genetic data and robust MR methods. Our results suggest a potential causal relationship between increased DPP4 gene expression at the mRNA level and HF in European populations. Although the estimated OR is modest and the CIs vary across methods, the triangulation of evidence increases confidence in this association. This triangulation encompasses RAPS-based methods (MR-RAPS, GRAPPLE, and BESIDE-MR) and debiased IVW, both of which can incorporate weak and strong IVs, as well as classical IVW using genome-wide significant IVs. Debiased IVW showed limited evidence for a causal effect of DPP4 inhibition on CVDs, which may be attributable to low statistical power. Overall, our findings suggest that the effect of DPP4 inhibitors on HF, which is mediated by DPP4 gene expression at the mRNA level, is unlikely to be harmful. There was limited evidence to support that DPP4 inhibitors have an indirect effect mediated by DPP4 gene expression at the mRNA level on other CVDs. Several clinical trials have explored the cardiovascular safety of DPP4 inhibitors, yet their findings remain inconsistent. For example, the SAVOR-TIMI 53 trial reported a 27% increased risk of HF hospitalization with saxagliptin [6–8], which aligns with our observation that increased DPP4 expression is causally linked to HF. In contrast, the TECOS trial with sitagliptin did not demonstrate a significant increase in HF-related outcomes [9, 10]. These discrepancies may stem from differences in the pharmacological properties of the agents, variations in patient populations, and differences in study designs. Our MR approach complements these clinical findings by providing genetic evidence that elevated DPP4 expression contributes to HF pathogenesis—potentially through mechanisms such as inflammation and adverse cardiac remodeling—while DPP4 inhibition itself does not exert an indirect detrimental effect on other CVDs.

The biological mechanisms underlying increased DPP4 expression in HF are not fully elucidated; however, existing evidence implicates DPP4 in key pathological processes. DPP4 may contribute to myocardial fibrosis by promoting stromal cell activation and extracellular matrix deposition [41], and it may exacerbate chronic inflammation through modulation of chemokines and cytokines [42]. Additionally, DPP4 degrades protective neuropeptides such as GLP-1, whose cardioprotective effects on metabolism and contractility are well established [43]. Moreover, experimental studies have shown that DPP4 inhibition can ameliorate diet-induced cardiac dysfunction and oxidative stress, further supporting a mechanistic link between DPP4 activity and HF progression [44]. These findings support a mechanistic link between elevated DPP4 expression and the progression of HF. Type 2 diabetes is a major cause of CVD, and recently, DPP4 inhibitors have been found to have potentially favorable effects in treating patients with CVDs [45]. Elevated plasma DPP4 activity in individuals without health conditions is a standalone predictor of the development of metabolic syndrome [46] and might constitute a pivotal link connecting central obesity, insulin resistance, and subsequent CVD [45]. Many studies have demonstrated the benefits of DPP4 inhibitors on CVD [47]. Chen et al. found that DPP4 inhibitors and GLP-1 receptor agonists (GLP-1 RAs) improved exercise tolerance in people with HF [48]. However, although the SAVOR-TIMI trial reported an increase in hospitalization for HF [8], an increased risk of HF was not observed in the Examination of Cardiovascular Outcomes with Alogliptin versus Standard of Care (EXAMINE) study, the Trial Evaluating Cardiovascular Outcomes with Sitagliptin (TECOS), or the Vildagliptin in Ventricular Dysfunction Diabetes (VIVIDD) trial [9–12, 49]. Additionally, a systematic review of randomized controlled studies indicated that targeting the DPP4-GLP-1 pathway improves exercise tolerance in people with HF [48].

We used two-sample MR methods to mimic the random group assignment process to clarify the causality between DPP4 gene expression at the mRNA level and CVD in people of European ancestry. Previously, MR methods have been used to investigate the causal relationship of circulating protein biomarkers on ischemic stroke and the impact of angiopoietin-like protein 3 (ANGPTL3) and 4 (ANGPTL4) inhibition of lipoprotein lipase (LPL) on CVD [50]. Our findings, leveraging MR-RAPS, GRAPPLE, and BESIDE-MR, highlight a potential increase in HF risk with elevated DPP4 gene expression at the mRNA level. The inhibition of DPP4 drug targets is unlikely to affect the risk of HF in European populations. Therefore, DPP4 inhibitors are not likely to cause HF directly, and the association between saxagliptin and HF may need an alternative explanation.

Our findings also clarify the causal mechanisms of DPP4 gene expression at the mRNA level on other CVDs, such as AF, MI, and stroke. First, no genetic heterogeneity was detected by GRAPPLE for AF, and results show that DPP4 gene expression at the mRNA level has no causality on AF. Moreover, this study indicates that DPP4 does not play a role in MI and that an increased TC level may increase the risk of MI. This is consistent with the results of the Copenhagen General Population Study [51]. GRAPPLE analysis also identified three potential pleiotropic pathways linked to SLC4A10, BMI, and TC. Also, the PPI of BESIDE-MR suggests that rs12619850 on SLC4A10 and rs635634 related to TC may not be suitable as IVs. Thus, SLC4A10, BMI, and TC may be related to correlated pleiotropic effects. This aligns with previous observational studies that showed that DPP4 could impact body weight, central fat distribution, lipid profile, and BMI in postmenopausal women [52–54]. The DPP4 locus is flanked by the GCG gene (encoding proglucagon, the source of glucagon, GLP-1, and GLP-2) and the SLC4A10 gene, both of which have some shared characteristics, such as involvement in neurological disorders [55, 56]. Therefore, a multivariate GRAPPLE analysis was conducted with adjustment for these factors, and the results showed that DPP4 gene expression at the mRNA level did not affect the risk of MI. BESIDE-MR also gave the same results. We conducted similar analyses for stroke, which produced comparable results. The results from the MR estimates suggest that DPP4 gene expression at the mRNA level has no causal relationship with AF, MI, and stroke.

Comparative Analysis with Other Glucose-Lowering Therapies: Our study's finding of no significant causal association between DPP4 gene expression/inhibition and AF contrasts with the documented benefits of SGLT2 inhibitors and GLP-1 receptor agonists on AF outcomes [53]. These mechanistic differences imply that while elevated DPP4 expression may contribute to HF via pathways such as inflammation and adverse cardiac remodeling, it does not appear to affect AF in the same manner. A similar comparative analysis is observed in the context of MI, where recent insights into SGLT2 inhibitors indicate divergent effects compared to DPP4 modulation [54]. Further research is warranted to elucidate the specific pathways underlying these differences.

The main strength of the present study includes using a series of MR that can utilize the strong and weak IVs to infer the causality of DPP4 gene expression at the mRNA level/DPP4 inhibition on CVD. This approach reduces the selection bias since the standard practice in MR is only to use the significant SNPs as IVs. In addition, we performed sensitivity analyses to account for biases resulting from weak IV and pervasive pleiotropy. However, this study also has limitations. Although our MR method is permissive for weak IV, the number of cases may have been insufficient. The outcome and mediator data, including CVD and type 2 diabetes mellitus, were obtained from significant biobanks and genetic studies, such as the UKB and the FinnGen project, ensuring the statistical power of the study's findings and conclusions. Second, while debiased IVW can handle weak IVs, the limited number of genome-wide significant SNPs associated with DPP4 inhibition substantially reduces statistical power and increases susceptibility to weak IV bias. This limitation affects our ability to make strong inferences regarding the effects of DPP4 inhibition and underscores the potential for underestimating even modest causal effects, necessitating caution in our interpretations. Our study employed two distinct strategies to evaluate the potential effects of DPP4 inhibition: (1) using cis-eQTLs for DPP4 gene expression at the mRNA level in peripheral blood and (2) using HbA1c-associated variants to approximate the downstream glycemic effects of DPP4 inhibitor therapy. Each approach has inherent limitations. For the expression-based approach, although cis-eQTLs provide a biologically plausible proxy for DPP4 inhibition, they may not fully capture enzymatic activity or pharmacological inhibition. Notably, we did not include known loss-of-function variants in DPP4, which would more directly mimic the mechanism of inhibition. Moreover, expression measurements from peripheral blood may not fully represent DPP4 activity in metabolically relevant tissues. For the glycemic trait–based approach, our use of the term genetically proxied for DPP4 inhibition refers explicitly to HbA1c-lowering variants. While this approach aligns with established drug-target MR frameworks, such as those proposed by Walker et al. [18] and Zheng et al. [57], these variants may influence cardiovascular outcomes via pleiotropic pathways unrelated to DPP4. A recent commentary [58] has also emphasized that genetic proxies based on glycemic biomarkers (such as HbA1c) represent the downstream biomarker-lowering effects of drug targets, not direct analogs of pharmacologic inhibition. This distinction highlights that glycemic trait–based instruments may not fully capture the biological specificity of DPP4 inhibition and could reflect broader metabolic influences, thereby complicating interpretation.

In defining the DPP 4 inhibition process (Method S1 in Supporting 1), the primary source was the 48 best SNPs from GTEx, from which we then further refined suitable SNPs as genetic proxies using HbA1c as a criterion, and finally, these SNPs were then screened under IV conditions. However, even if we tried to use a relaxed LD criterion such as 0.8 in the aggregation threshold in addition to the original criterion, there is a limited number of genetic variants associated with DPP4 inhibition, which may reduce the statistical power to detect causal associations. This constraint limits our ability to robustly assess the direct effects of DPP4 inhibition on CVD independent of type 2 diabetes mellitus (the IV number can be used in multi-GRAPPLE is less than 2). Future studies with larger genetic datasets or alternative methodologies may be needed to validate these findings and improve the precision of causal estimates. Additionally, we examined four cardiovascular outcomes based on a priori biological and clinical relevance, without designating a single primary endpoint. Among these, only the association between DPP4 expression and HF reached nominal statistical significance (approximately *p*=0.014). Although this result would not withstand a strict Bonferroni correction for four comparisons, the consistent findings across multiple pleiotropy-robust MR methods lend support to its validity.

Beyond transcript-level analyses, future studies may consider incorporating MR based on protein quantitative trait loci (pQTL) for circulating DPP4 protein levels. Since mRNA expression does not always correlate directly with protein abundance or enzymatic activity, pQTL-based MR could provide complementary insights into the causal role of DPP4 at the protein level in cardiovascular and metabolic diseases. Moreover, pQTL data derived from ancestrally diverse cohorts and larger sample sizes would help refine the biological understanding and increase the generalizability of MR findings. This integrative approach may help triangulate evidence from multiple molecular layers, thereby improving the translational relevance of MR studies in this field.

Finally, our study was restricted to individuals of European ancestry, which limits the generalizability of our findings to other populations. Population differences in allele frequencies and LD structures may affect the strength and validity of genetic instruments, thereby potentially limiting the transferability of MR results across ancestries [59]. Furthermore, pharmacogenomic differences across ethnic groups are well documented, particularly in the context of DPP4 inhibitor responses. For instance, studies conducted in Japanese populations have demonstrated that DPP4 inhibitor use may be associated with a lower risk of first cardiovascular events and HF compared to Western populations [15, 16]. These findings highlight the possibility of ethnic heterogeneity in pharmacologic response, and underscore the need for future MR studies in more diverse ancestral groups to confirm the external validity of our results.

## 5. Conclusion

Our findings, based on a series of robust to weak IVs and genetic heterogeneity MR analyses, suggest that increased DPP4 gene expression at the mRNA level may be causally linked to HF but not to AF, MI, or stroke in individuals of European ancestry. This association appears unlikely to be mediated by type 2 diabetes mellitus. By contrast, there was limited evidence for a causal relationship between genetically proxied DPP4 inhibition and cardiovascular outcomes, a result that may reflect low statistical power rather than a genuine absence of effect. Additionally, if DPP4 inhibition affects cardiovascular outcomes, they may not do so through glycemic control, such as HbA1c reduction. Taken together, these findings suggest that, in European populations, DPP4 inhibitors are unlikely to have a detrimental effect on HF via DPP4 gene expression at the mRNA level; however, there is no evidence to support a causal role in other cardiovascular outcomes.

## Figures and Tables

**Table 1:** The MR results of the causal estimates of DPP4 gene expression at the mRNA level on HbA1c.

MR methods	Estimate	Standard error (SE)	*p* value	CI
MR-RAPS	0.017	0.071	8.09*E* − 01	(−0.122, 0.157)
GRAPPLE	0.018	0.072	8.03*E* − 01	(−0.123, 0.158)
BESIDE-MR (DL)	0.024	0.056	—	(−0.081, 0.145)
BESIDE-MR (Bayesian)	0.079	0.064	—	(−0.045, 0.207)
Debiased IVW	−0.053	0.077	4.94*E* − 01	(−0.204, 0.098)

**Table 2:** The MR results for DPP4 inhibition on CVD based on GTEx.

Outcomes	dIVW estimate	Standard error (SE)	95% CI	*p* value	Condition
All-cause heart failure	−0.801	0.548	(−1.876, 0.273)	0.144	74.76
Atrial fibrillation	0.676	0.424	(−0.155, 1.506)	0.111	74.76
Myocardial infarction	1.062	1.932	(−2.724, 4.848)	0.583	74.76
Stroke	−0.25	1.674	(−3.530, 3.030)	0.881	74.76

## Data Availability

The data that support the findings of this study are available upon request from the corresponding author. The data are not publicly available due to privacy or ethical restrictions.

## Supporting Information

Supporting Information 1 Supporting 1.

Figure S1. Study design for DPP4 gene expression at the mRNA level.

Figure S2. Study design.

Method S1. Study design for DPP4 inhibition.

Method S2. Description of the eQTLGen, UK Biobank, FinnGen, and GTEx.

Table S1. STROBE-MR Checklist.

Table S2. Information about the GWAS summary datasets.

Table S3. Definitions of disease phenotype for UK Biobank.

Method S3. Introduction of MR-RAPS, GRAPPLE, and BESIDE-MR.

Method S4. Introduction of the debiased inverse-variance weighted estimator.

Method S5. The causal mediation analysis by MR.

Table S4. The criterion of the selection *p* value and the number of SNPs in MR.

Method S6. The diagnostic plots for MR-RAPS.

Table S5. Baseline characteristics of the participants.

Method S7. Identified potential pleiotropic pathways.

Figure S3. The odds ratio of the DPP4 gene expression at the mRNA level on CVD via univariate MR.

Method S8. The multivariate GRAPPLE for the pathway from DPP4 gene expression at the mRNA level to CVD.

Supporting Information 2 Supporting 2.

Table S1. Selected SNPs for univariate MR: DPP4 gene expression at the mRNA level on HF.

Table S2. Selected SNPs for univariate MR: DPP4 gene expression at the mRNA level on AF.

Table S3. Selected SNPs for univariate MR: DPP4 gene expression at the mRNA level on MI.

Table S4. Selected SNPs for univariate MR: DPP4 gene expression at the mRNA level on stroke.

Table S5. Details of SNPs for mode 1 and related genes and phenotypes for MR: DPP4 gene expression at the mRNA level on MI.

Table S6. Details of SNPs for mode 2 and related genes and phenotypes for MR: DPP4 gene expression at the mRNA level on MI.

Table S7. Details of SNPs for mode 3 and related genes and phenotypes for MR: DPP4 gene expression at the mRNA level on MI.

Table S8. Details of SNPs for mode 1 and related genes and phenotypes for MR: DPP4 gene expression at the mRNA level on stroke.

Table S9. Details of SNPs for mode 2 and related genes and phenotypes for MR: DPP4 gene expression at the mRNA level on stroke.

Table S10. Selected SNPs for multivariate MR: DPP4 gene expression at the mRNA level, SLC4A10, BMI, and TC on MI.

Table S11. Selected SNPs for multivariate MR: DPP4 gene expression at the mRNA level, HTN, and TG on stroke.

Table S12. PPI in the valid instrument set for SNP for the effect of DPP4 gene expression at the mRNA level on HF.

Table S13. PPI in the valid instrument set for SNP for the effect of DPP4 gene expression at the mRNA level on AF.

Table S14. PPI in the valid instrument set for SNP for the effect of DPP4 gene expression at the mRNA level on MI.

Table S15. PPI in the valid instrument set for SNP for the effect of DPP4 gene expression at the mRNA level on stroke.

Table S16. The one-component BESIDE-MR with penalization for DPP4 gene expression at the mRNA level on HF.

Table S17. The one-component BESIDE-MR with penalization for DPP4 gene expression at the mRNA level on AF.

Table S18. The one-component BESIDE-MR with penalization for DPP4 gene expression at the mRNA level on MI.

Table S19. The one-component BESIDE-MR with penalization for DPP4 gene expression at the mRNA level on stroke.

Table S20. The results of classical IVW for DPP4 gene expression at the mRNA level on CVD.

Table S21. The results of debiased IVW for DPP4 gene expression at the mRNA level on CVD.

Table S22. Selected SNPs for the mediation analysis: DPP4 gene expression at the mRNA level, type 2 diabetes, and HF.

Table S23. Selected SNPs for the mediation analysis: DPP4 gene expression at the mRNA level, type 2 diabetes, and AF.

Table S24. Selected SNPs for the mediation analysis: DPP4 gene expression at the mRNA level, type 2 diabetes, and MI.

Table S25. Selected SNPs for the mediation analysis: DPP4 gene expression at the mRNA level, type 2 diabetes, and stroke.

Table S26. Selected SNPs for univariate MR: DPP4 gene expression at the mRNA level on HbA1c.
