# Supplementary material for: The Causal Mechanism Between the Dipeptidyl Peptidase-4, Heart Failure, and Other Cardiovascular Diseases: A Mendelian Randomization and Mediation Study
Source: Int J Endocrinol. 2025 Aug 26;2025:2357272. doi: 10.1155/ije/2357272 (PMC12404834; doi:10.1155/ije/2357272)
Supplement: Supporting Information 1 — Supporting 1. [file 2357272.f1.docx]

**The causal mechanism between the dipeptidyl peptidase-4, heart failure, and other cardiovascular diseases: A Mendelian randomization and mediation study**

Che-Kai Chen, PhD; Chang-Fu Kuo, MD, PhD; Yu-Jing Chang, MSc; Weiya Zhang, PhD; Michael Doherty, MD; Ming-Ling Chang, MD, PhD; Tsung-Hsing Chen, MD, PhD

**Supplement 1 Content**

**Figure S1.** Study design for DPP4 gene expression at the mRNA level……………………………………………….…3

**Figure S2.** Study design………………………………………………………………………………………………………………………..4

**Method S1.** Study design for DPP4 inhibition ……………………………………………………………………………………..5

**Method S2.** Description of the eQTLGen, UK Biobank, FinnGen, and GTEx ……………………………………….19

**Table S1.** STROBE-MR Checklist …………………………………………………………………………………………………….....21

**Table S2.** Information about the GWAS summary datasets ……………………………………………………………...43

**Table S3.** Definitions of disease phenotype for UK Biobank …………………………………………………………..…45

**Method S3.** Introduction of MR-RAPS, GRAPPLE, and BESIDE-MR …………………………………………………...46

**Method S4.** Introduction of the debiased inverse-variance weighted estimator………………………..……..50

**Method S5.** The causal mediation analysis by MR ………………………………………………………………………..….51

**Table S4.** The criterion of the selection p-value and the number of SNPs in MR …………………………..….53

**Method S6.** The diagnostic plots for MR-RAPS ……………………………………………………………………………..….54

**Table S5.** Baseline characteristics of the participants ……………………………………………………………………….57

**Method S7.** Identified potential pleiotropic pathways…………………………...…………………………………….…..58

**Figure S3.** The odds ratio of the DPP4 gene expression at the mRNA level on CVD via univariate MR ….………………………………………………………………..…………………………………………………………..61

**Method S8.** The multivariate GRAPPLE for the pathway from DPP4 gene expression at the mRNA level

to CVD …………………………………………………………………………………………………………………………..62

**References** ……………………………………………………………………………………………………………………………………..69

**Figure S1. Study design** **for DPP4 gene expression at the mRNA level**

**
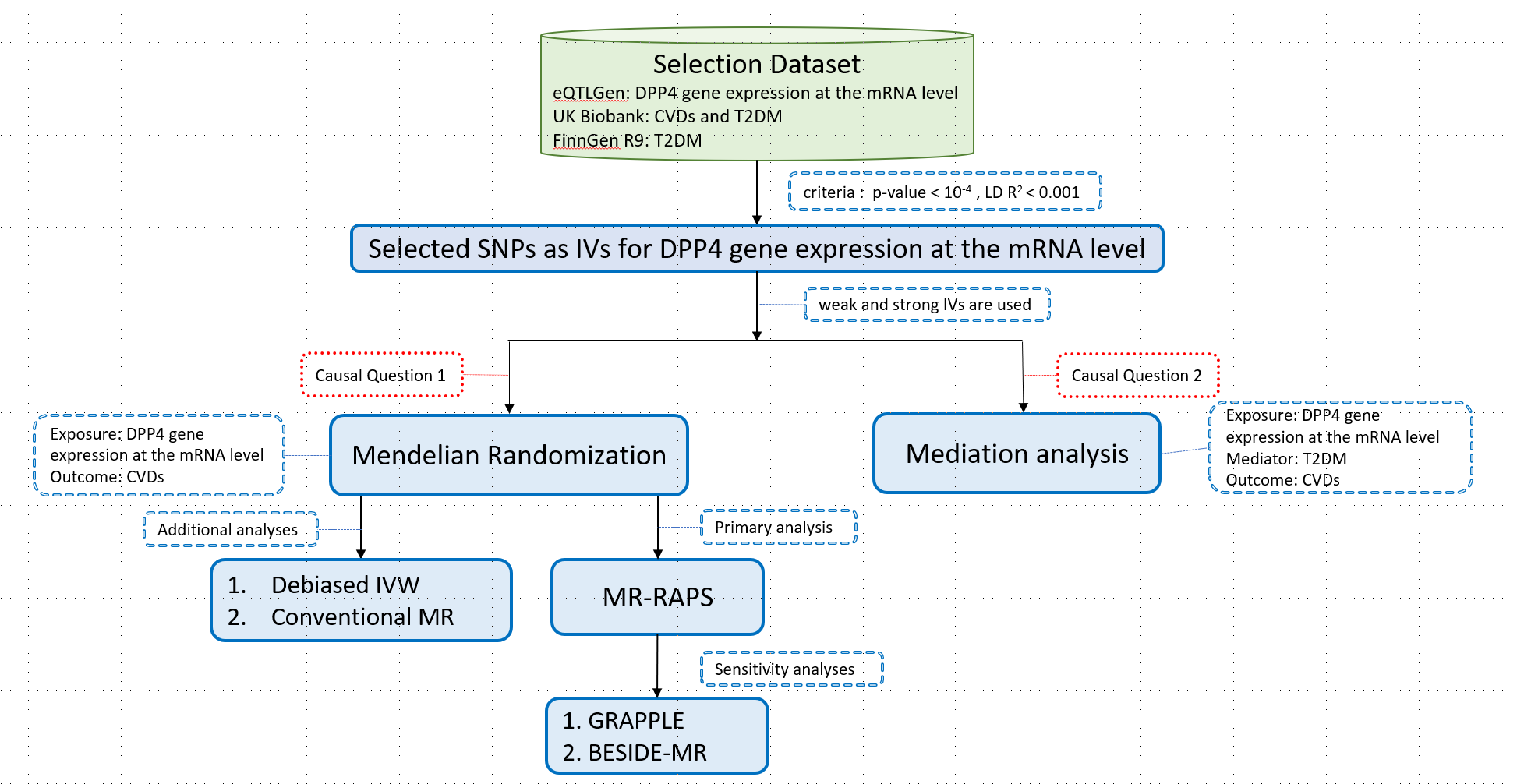
**

Causal questions: 1) Does the DPP4 gene expression at the mRNA level play a causal role in CVD, including HF, AF, MI, and stroke? 2) Does the DPP4 gene expression at the mRNA level directly affect CVD independently of the mediator type 2 diabetes mellitus?

**Figure S2. Study design**


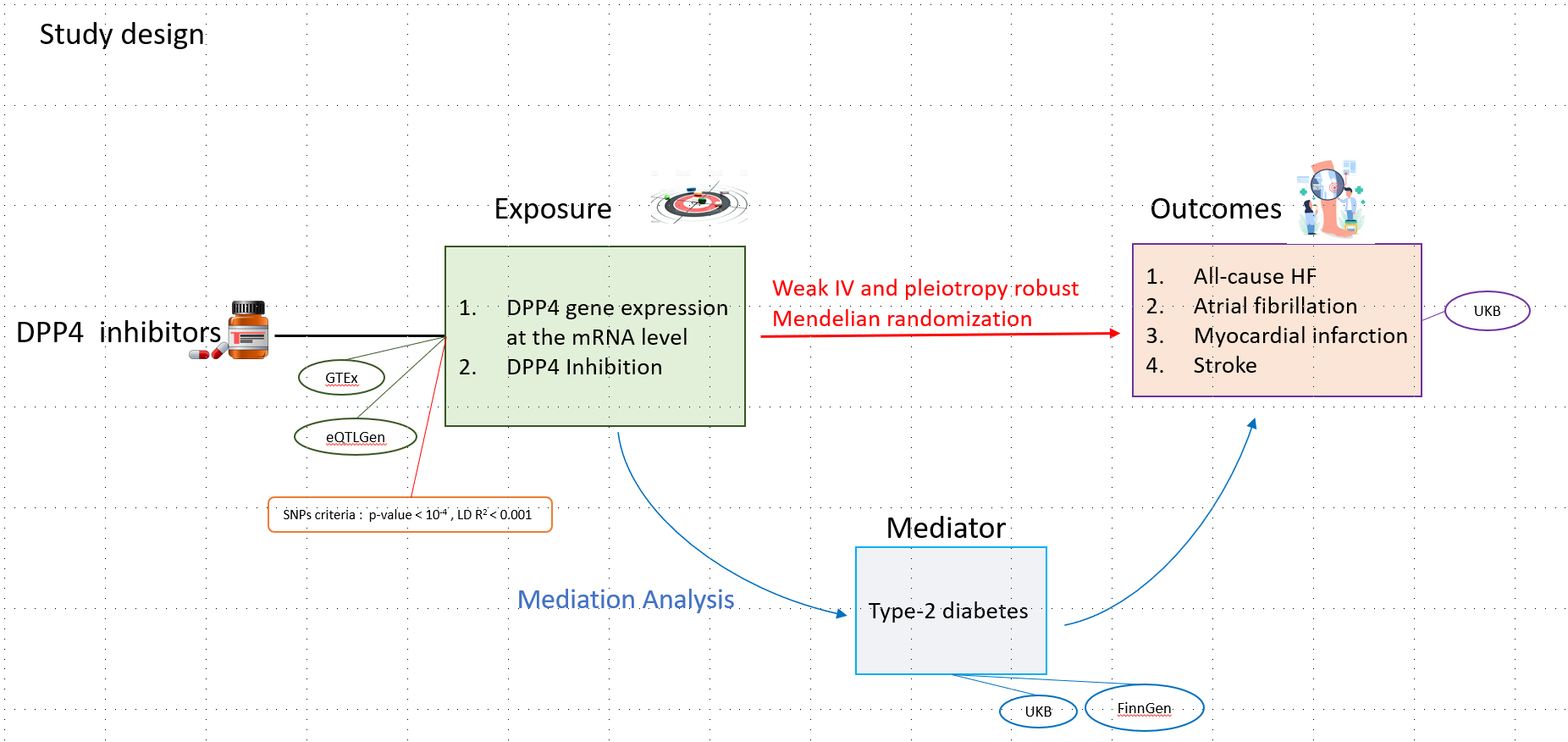


We addressed clinically pertinent queries framed in a causal context: (1) Does DPP4 gene expression at the mRNA level exert a causal influence on the development of CVD? (2) Is the impact of DPP4 gene expression at the mRNA level on CVD independent of the mediation by type-2 diabetes? (3) Is there a causal association between DPP4 inhibition and the onset of CVDs?

**Method S1. Study design for DPP4** **inhibition**

The following study was designed to explore the causality of dipeptidyl peptidase-4 (DPP4) inhibition on cardiovascular disease (CVDs) and type 2 diabetes mellitus, which used a similar idea as the work by Venexia et al.[1] and Xu Min et al.[2]. We divided our study design into two parts to elaborate on it. First, we explain how to define the new exposure DPP4 inhibition. Then, we employ debiased IVW, which is more robust to weak IV and balanced horizontal pleiotropy than the previous two studies, to assess the causal effect of DPP4 inhibition on CVDs. We will describe the process and results separately according to DPP4 eQTL data from the GTEx dataset or eQTLGen Consortium.

1. **GTEx dataset**

**Part I. Defined** **DPP4 inhibition:**

**Step 1.** Collected the best SNP in the DPP4 gene:

We collected the best SNP in each tissue in GTEx (GTEx_Analysis_v8_eQTL)[3] for the DPP4 gene with gene ID ENSG00000197635.9 (eTable A1). In this step, we have 48 'best SNPs' and the effect of the DPP4 Gene on each SNP$(G_{j}$), $\beta_{1,j}$ with its p-value of $\beta_{1,j}$(nominal p-value) denoted by $P_{1,j}$ which depends on SNP $G_{j}$ (eTable 1). The 'best SNP' represents the variant with the smallest nominal p-value for a variant-gene pair in GTEx in the eGenes association file.

**Step 2.** Find the effect of the DPP4 Gene on HbA1c induced by the best SNP:

We collected GWAS data for HbA1c from the OpenGWAS project. Then we used each SNP as IV and conducted the MR (debiased IVW) to derive the effect of the DPP4 Gene on HbA1c, saying "$\beta_{2,j}$" with its p-value $P_{2,j}$. These results are presented in eTable A2. Notice that these effects are different, which depends on the association of each SNP on HbA1c, and the number of SNPs decreases because there is no corresponding SNP data for HbA1.

**Step 3.** Given the definition of DPP4 inhibition based on the preceding steps:

To define the new exposure DPP4 inhibition, we first define $\beta_{j}=\beta_{1,j}\cdot\beta_{2,j}$, which means the effect of each best SNP on HbA1c through the DPP4 Gene, in which its p-value of beta $P_{j}=max\{P_{1,j},P_{2,j}\}$ based on the joint significance test and its standard error $\sigma_{\beta,j}$ approximately by

$$\sigma_{\beta,j}\approx\sqrt{\beta_{1,j}^{2}\sigma_{2,j}^{2}+\beta_{2,j}^{2}\sigma_{1,j}^{2}}$$

Here, we have neglected the covariance of $\beta_{1,j}$ and $\beta_{2,j}$ in the above approximation because our summarized data lacks this information. Furthermore, this step also excluded these best SNP with the $\beta_{j}\geq0$ and $P_{j}\geq0.05.$ Now, we can define the new exposure "DPP4 inhibition", which is related to these SNPs and satisfies the association of DPP4 inhibition on these SNP $G_{j}$ is equal $\beta_{j}<0$ with standard error $\sigma_{\beta,j}$ and p-value $P_{j}$ (eTable A3).

**eTable A1.** For DPP4 Gene, the best SNP in each tissue in GTEx

| Tissue | Best SNP $G_{j}$ | Effect Allele | Other Allele | Beta $\beta_{1,j}$ | Standard Error $\sigma_{1,j}$ | P-value $P_{1,j}$ |
| --- | --- | --- | --- | --- | --- | --- |
| Adipose_Subcutaneous | rs34536659 | G | C | -0.070 | 0.024 | 3.10E-03 |
| Adipose_Visceral_Omentum | rs79077660 | C | T | 0.457 | 0.130 | 4.66E-04 |
| Adrenal_Gland | rs10185053 | A | G | 0.577 | 0.060 | 3.86E-18 |
| Artery_Aorta | rs73971191 | T | C | 0.731 | 0.174 | 3.54E-05 |
| Artery_Coronary | rs75053379 | G | A | -0.740 | 0.179 | 5.49E-05 |
| Artery_Tibial | rs74698413 | G | A | 0.253 | 0.071 | 3.93E-04 |
| Brain_Amygdala | rs148313349 | CCTT | C | -1.553 | 0.492 | 2.07E-03 |
| Brain_Anterior_cingulate_cortex_BA24 | rs305679 | G | A | -0.399 | 0.108 | 3.23E-04 |
| Brain_Caudate_basal_ganglia | rs139780345 | C | T | 0.982 | 0.291 | 9.22E-04 |
| Brain_Cerebellar_Hemisphere | rs10170377 | G | A | -0.405 | 0.127 | 1.75E-03 |
| Brain_Cerebellum | rs115235187 | T | C | 0.689 | 0.202 | 8.07E-04 |
| Brain_Cortex | rs2080714 | G | T | 0.229 | 0.052 | 2.09E-05 |
| Brain_Frontal_Cortex_BA9 | rs114368432 | G | A | -0.506 | 0.122 | 6.00E-05 |
| Brain_Hippocampus | rs149242887 | C | T | -1.606 | 0.377 | 3.97E-05 |
| Brain_Hypothalamus | rs34869697 | G | A | 1.508 | 0.483 | 2.19E-03 |
| Brain_Nucleus_accumbens_basal_ganglia | rs143151639 | A | G | -1.571 | 0.378 | 5.25E-05 |
| Brain_Spinal_cord_cervical_c-1 | rs1435007 | G | A | -0.754 | 0.189 | 1.30E-04 |
| Brain_Substantia_nigra | rs368177604 | G | T | -0.683 | 0.191 | 5.47E-04 |
| Breast_Mammary_Tissue | rs73013291 | A | G | -0.595 | 0.164 | 3.37E-04 |
| Cells_Cultured_fibroblasts | rs79950401 | A | G | 0.224 | 0.058 | 1.33E-04 |
| Cells_EBV-transformed_lymphocytes | rs76608255 | T | C | 0.611 | 0.198 | 2.48E-03 |
| Colon_Sigmoid | rs6733162 | G | C | -0.206 | 0.059 | 5.51E-04 |
| Colon_Transverse | rs873826 | G | A | 0.316 | 0.059 | 1.52E-07 |
| Esophagus_Gastroesophageal_Junction | rs147641773 | T | C | 0.826 | 0.246 | 9.16E-04 |
| Esophagus_Mucosa | rs4616494 | A | G | 0.133 | 0.040 | 1.01E-03 |
| Esophagus_Muscularis | rs560995114 | TAGTA | T | 0.756 | 0.195 | 1.21E-04 |
| Heart_Atrial_Appendage | rs55727854 | T | C | -0.252 | 0.072 | 5.15E-04 |
| Heart_Left_Ventricle | rs2075340 | A | T | -0.218 | 0.054 | 5.88E-05 |
| Kidney_Cortex | rs79961351 | A | G | 0.677 | 0.210 | 2.29E-03 |
| Liver | rs56413500 | G | A | -0.264 | 0.086 | 2.40E-03 |
| Lung | rs75166367 | G | A | 0.435 | 0.067 | 2.95E-10 |
| Minor_Salivary_Gland | rs188581353 | G | A | 1.149 | 0.272 | 4.77E-05 |
| Muscle_Skeletal | rs74965424 | C | T | 0.284 | 0.089 | 1.58E-03 |
| Nerve_Tibial | rs78618724 | C | T | 0.820 | 0.221 | 2.28E-04 |
| Ovary | rs148183493 | CACA | C | 0.509 | 0.132 | 1.78E-04 |
| Pancreas | rs62189692 | G | C | 0.209 | 0.056 | 2.11E-04 |
| Pituitary | rs74729137 | C | T | -0.688 | 0.233 | 3.58E-03 |
| Prostate | rs75850673 | G | T | 0.167 | 0.048 | 5.75E-04 |
| Skin_Not_Sun_Exposed_Suprapubic | rs2111850 | T | C | 0.120 | 0.032 | 2.12E-04 |
| Skin_Sun_Exposed_Lower_leg | rs10165813 | C | T | 0.215 | 0.059 | 3.01E-04 |
| Small_Intestine_Terminal_Ileum | rs145146665 | GTAAAC | G | 0.647 | 0.187 | 7.20E-04 |
| Spleen | rs17759529 | C | G | -0.265 | 0.079 | 9.06E-04 |
| Stomach | rs75479124 | C | A | 0.701 | 0.219 | 1.53E-03 |
| Testis | rs9287809 | G | A | -0.558 | 0.177 | 1.79E-03 |
| Thyroid | rs4233648 | C | T | 0.175 | 0.037 | 3.68E-06 |
| Uterus | rs1551053 | T | G | -1.079 | 0.278 | 1.85E-04 |
| Vagina | rs78228296 | T | C | -1.189 | 0.340 | 6.60E-04 |
| Whole_Blood | rs79691504 | G | A | -0.263 | 0.085 | 2.01E-03 |

**eTable A2.** The MR (debiased IVW) results of the effect of DPP4 Gene on HbA1c as each SNP as IV.

| SNP $G_{j}$ | $\beta_{2,j}$ | Standard Error $\sigma_{2,j}$ | CI_lower | CI_upper | P-value $P_{2,j}$ | Condition |
| --- | --- | --- | --- | --- | --- | --- |
| rs10185053 | -0.003 | << 0.01 | -0.003 | -0.003 | << 5E-8 | 91.5 |
| rs114368432 | 0.019 | 0.002 | 0.015 | 0.022 | 2.84E-30 | 16.2 |
| rs115235187 | -0.014 | 0.002 | -0.017 | -0.010 | 5.28E-14 | 10.6 |
| rs139780345 | -0.032 | 0.004 | -0.040 | -0.023 | 1.75E-13 | 10.4 |
| rs143151639 | -0.033 | 0.003 | -0.038 | -0.027 | 1.31E-30 | 16.3 |
| rs1435007 | 0.007 | 0.001 | 0.006 | 0.008 | 8.88E-26 | 14.8 |
| rs147641773 | 0.022 | 0.003 | 0.016 | 0.028 | 4.64E-13 | 10.2 |
| rs148313349 | -0.003 | 0.000 | -0.004 | -0.002 | 2.13E-10 | 9.0 |
| rs149242887 | 0.009 | 0.001 | 0.008 | 0.010 | 7.28E-34 | 17.2 |
| rs1551053 | 0.023 | 0.002 | 0.018 | 0.027 | 3.22E-23 | 14.0 |
| rs17759529 | 0.027 | 0.004 | 0.020 | 0.035 | 2.24E-13 | 10.4 |
| rs188581353 | -0.025 | 0.002 | -0.030 | -0.021 | 1.27E-32 | 16.8 |
| rs2075340 | 0.008 | 0.001 | 0.007 | 0.010 | 2.99E-28 | 15.6 |
| rs2080714 | -0.025 | 0.002 | -0.029 | -0.021 | 6.59E-38 | 18.2 |
| rs2111850 | 0.134 | 0.015 | 0.105 | 0.163 | 5.42E-20 | 12.9 |
| rs305679 | 0.005 | 0.001 | 0.004 | 0.006 | 2.74E-19 | 12.7 |
| rs34536659 | 0.119 | 0.021 | 0.077 | 0.161 | 3.03E-08 | 7.8 |
| rs368177604 | 0.032 | 0.004 | 0.024 | 0.039 | 4.95E-17 | 11.9 |
| rs4233648 | -0.019 | 0.001 | -0.022 | -0.017 | 1.83E-49 | 20.9 |
| rs4616494 | -0.122 | 0.017 | -0.156 | -0.088 | 1.91E-12 | 10.0 |
| rs55727854 | 0.004 | 0.000 | 0.003 | 0.005 | 1.17E-15 | 11.3 |
| rs560995114 | -0.038 | 0.004 | -0.046 | -0.031 | 2.46E-23 | 14.1 |
| rs62189692 | -0.028 | 0.003 | -0.034 | -0.022 | 1.47E-20 | 13.1 |
| rs74698413 | 0.098 | 0.012 | 0.075 | 0.121 | 1.07E-16 | 11.7 |
| rs74729137 | -0.072 | 0.013 | -0.098 | -0.046 | 5.31E-08 | 7.7 |
| rs74965424 | -0.027 | 0.004 | -0.036 | -0.019 | 1.39E-10 | 9.1 |
| rs75166367 | -0.006 | 0.000 | -0.006 | -0.005 | 4.26E-181 | 40.6 |
| rs75850673 | -0.063 | 0.008 | -0.079 | -0.048 | 1.44E-15 | 11.3 |
| rs76608255 | 0.007 | 0.001 | 0.005 | 0.009 | 1.48E-09 | 8.6 |
| rs79077660 | -0.109 | 0.013 | -0.135 | -0.083 | 5.59E-16 | 11.5 |
| rs79691504 | -0.074 | 0.012 | -0.098 | -0.050 | 1.09E-09 | 8.6 |
| rs79950401 | 0.065 | 0.007 | 0.052 | 0.078 | 1.03E-22 | 13.9 |
| rs79961351 | 0.016 | 0.002 | 0.011 | 0.021 | 3.30E-11 | 9.4 |
| rs873826 | -0.024 | 0.001 | -0.027 | -0.022 | 8.04E-87 | 27.9 |

**eTable A3.** The related SNP to DPP4 inhibition

| SNPs | Effect Allele | Other Allele | $\beta_{j}$ | Standard Error$\sigma_{\beta,j}$ | P-value $P_{j}$ |
| --- | --- | --- | --- | --- | --- |
| rs10185053 | A | G | -0.002 | 0.0002 | 3.86E-18 |
| rs114368432 | G | A | -0.009 | 0.0024 | 6.00E-05 |
| rs115235187 | T | C | -0.009 | 0.0030 | 8.07E-04 |
| rs139780345 | C | T | -0.031 | 0.0101 | 9.22E-04 |
| rs1435007 | G | A | -0.005 | 0.0014 | 1.30E-04 |
| rs149242887 | C | T | -0.014 | 0.0036 | 3.97E-05 |
| rs1551053 | T | G | -0.024 | 0.0067 | 1.85E-04 |
| rs17759529 | C | G | -0.007 | 0.0024 | 9.06E-04 |
| rs188581353 | G | A | -0.029 | 0.0073 | 4.77E-05 |
| rs2075340 | A | T | -0.002 | 0.0005 | 5.88E-05 |
| rs2080714 | G | T | -0.006 | 0.0014 | 2.09E-05 |
| rs305679 | G | A | -0.002 | 0.0005 | 3.23E-04 |
| rs34536659 | G | C | -0.008 | 0.0032 | 3.10E-03 |
| rs368177604 | G | T | -0.022 | 0.0066 | 5.47E-04 |
| rs4233648 | C | T | -0.003 | 0.0008 | 3.68E-06 |
| rs4616494 | A | G | -0.016 | 0.0054 | 1.01E-03 |
| rs55727854 | T | C | -0.001 | 0.0003 | 5.15E-04 |
| rs560995114 | TAGTA | T | -0.029 | 0.0080 | 1.21E-04 |
| rs62189692 | G | C | -0.006 | 0.0017 | 2.11E-04 |
| rs74965424 | C | T | -0.008 | 0.0027 | 1.58E-03 |
| rs75166367 | G | A | -0.002 | 0.0004 | 2.95E-10 |
| rs75850673 | G | T | -0.011 | 0.0033 | 5.75E-04 |
| rs79077660 | C | T | -0.050 | 0.0154 | 4.66E-04 |
| rs873826 | G | A | -0.008 | 0.0015 | 1.52E-07 |

**Part II. Assess the causal effect of DPP4 inhibition on CVDs and type 2 diabetes:**

We extracted only SNPs with a weak LD with a correlation coefficient square R^2^ <0.001. It is worth noting that we are not setting screening criteria for p-values ​​here because debiased IVW is robust to weak IVs.

Now, we employ debiased IVW to assess the causal effect of DPP4 inhibition on CVDs. The selected SNPs as IVs and the MR results of DPP4 inhibition on CVDs can be found in eTables A4 and A5, respectively.

**eTable A4.** IVs for MR: DPP4 inhibition on CVD based on GTEx

| SNPs | Effect Allele | Other Allele | Beta $\beta_{j}$ | Standard Error$\sigma_{\beta,j}$ | P-value $P_{j}$ |
| --- | --- | --- | --- | --- | --- |
| rs10185053 | A | G | -0.002 | 0.00017 | 3.86E-18 |
| rs2080714 | G | T | -0.006 | 0.001373 | 2.09E-05 |

**eTable A5.** The MR results for DPP4 inhibition on CVD based on GTEx

| Outcomes | dIVW Estimate | Std Error | 95% CI | P-value | Condition |
| --- | --- | --- | --- | --- | --- |
| All-cause heart failure | -0.801 | 0.548 | (-1.876, 0.273) | 0.144 | 74.76 |
| Atrial fibrillation | 0.676 | 0.424 | (-0.155, 1.506) | 0.111 | 74.76 |
| Myocardial infarction | 1.062 | 1.932 | (-2.724, 4.848) | 0.583 | 74.76 |
| Stroke | -0.25 | 1.674 | (-3.530, 3.030) | 0.881 | 74.76 |

1. **eQTLGen Consortium**

We collected eQTL GWAS with 15300 SNPs in the eQTLGen Consortium, where we did not use the concept of the best SNPs. Then, we used the same process as in the GTEx dataset. Here, we report 18 selected SNPs related to DPP4 inhibition as IVs, and the MR results of DPP4 inhibition on CVDs can be found in eTables B1 and B2, respectively.

**eTable B1.** IVs for MR: DPP4 inhibition on CVD based on eQTLGen

| SNP | Effect Allele | Other Allele | $\beta_{j}$ | Standard Error$\sigma_{\beta,j}$ | P-value $P_{j}$ |
| --- | --- | --- | --- | --- | --- |
| rs10165200 | A | G | -0.001 | 0.0005 | 2.19E-03 |
| rs11587735 | C | A | -0.038 | 0.0176 | 9.94E-03 |
| rs11924390 | C | T | 0.000 | 0.0001 | 8.04E-03 |
| rs12413946 | C | T | -0.021 | 0.0090 | 7.05E-03 |
| rs12613025 | C | T | -0.006 | 0.0025 | 8.92E-03 |
| rs13292136 | T | C | -0.006 | 0.0024 | 4.52E-03 |
| rs1509123 | T | C | -0.028 | 0.0119 | 6.89E-03 |
| rs1511589 | A | G | -0.008 | 0.0035 | 8.45E-03 |
| rs17784882 | A | C | -0.014 | 0.0060 | 7.36E-03 |
| rs2145272 | G | A | -0.019 | 0.0080 | 5.36E-03 |
| rs3114018 | A | C | -0.003 | 0.0009 | 5.62E-04 |
| rs314370 | C | T | -0.020 | 0.0085 | 6.02E-03 |
| rs34977319 | T | C | -0.004 | 0.0012 | 1.87E-04 |
| rs4323050 | T | C | -0.068 | 0.0263 | 3.39E-03 |
| rs471364 | C | T | -0.006 | 0.0024 | 6.19E-03 |
| rs6583203 | C | T | -0.003 | 0.0011 | 3.27E-03 |
| rs72774609 | A | G | -0.021 | 0.0094 | 9.31E-03 |
| rs9820070 | C | A | -0.022 | 0.0103 | 9.43E-03 |

**eTable B2.** The MR results for DPP4 inhibition on CVD based on eQTLGen

| Outcomes | dIVW_estimate | Std Error | 95% CI | P-value | Condition |
| --- | --- | --- | --- | --- | --- |
| All-cause heart failure | -0.233 | 0.165 | (-0.556, 0.090) | 0.158 | 21.829 |
| Atrial fibrillation | -0.066 | 0.145 | (-0.349, 0.218) | 0.65 | 21.829 |
| Myocardial infarction | -0.289 | 0.192 | (-0.667, 0.088) | 0.13 | 21.829 |
| Stroke | -0.023 | 0.16 | (-0.337, 0.292) | 0.888 | 21.829 |

**Method S2. Description of the eQTLGen, UK Biobank, FinnGen, and GTEx**

- The eQTLGen Consortium ( <https://eqtlgen.org/index.html>), formed to identify downstream consequences of trait-related genetic variants, collected 31,684 blood samples, 10,317 trait-associated SNPs, 11M SNPs (MAF≧1%), and 19,942 genes to analyze in the cis-eQTL, trans-eQTL, and eQTS analysis. [4] Moreover, information about 37 participating cohorts with nearly European, contained in the eQTLGen Consortium, and the related analysis results can be found in Võsa et al.[4].
- The UK Biobank (UKB, <https://www.ukbiobank.ac.uk/>) is a large-scale biomedical database and research resource throughout England, Wales, and Scotland, which includes baseline data; questionnaire results; physical and cognitive test results; blood and urine samples; extensive environmental, lifestyle, and genetic data; and electronic health-related records for 502,448 patients aged 37-82. The two datasets do not overlap.
- FinnGen (<https://www.finngen.fi/en>) is a sizeable public-private partnership research project aiming to combine the imputed genetic data and health data from 500,000 Finnish biobank participants to provide new insight into the relationship between genetics and disease. On May 11, 2023, the latest GWAS summary statistic data of a total sample size of 377,277 (210,870 females and 166,407 males) were released, and the total number of variants 20,175,454 variants analyzed (Release 9, R9)[5].
- The Genotype-Tissue Expression (GTEx) project gathers non-diseased tissue samples from 1000 individuals across 54 sites for gene expression research. Data, including gene expression and histology images, is accessible through the GTEx Portal. The GTEx Project focuses on development-specific genetic impacts on gene expression in healthy neonatal, pediatric, and adolescent tissues, with a tissue bank and analysis resource.

**Table S1. STROBE-MR Checklist**

To report our findings explicitly, we followed the Strengthening the Reporting of Observational Studies in Epidemiology guidelines for Mendelian randomization (MR) [6]. Notice that in the following table, the page numbers indicated, unless otherwise specified, are all part of the Main manuscript and not in the Supplemental. The format of the STROBE-MR Checklist can be found on the STROBE-MR website (<https://www.strobe-mr.org/>).

| **Item No.** | **Section** | **Checklist item** | **Relevant paragraph location from manuscript or Supplemental** |
| --- | --- | --- | --- |
| 1 | TITLE and ABSTRACT | Indicate Mendelian randomization (MR) as the study's design in the title and/or the abstract if that is a main purpose of the study | This study used weak IV and pleiotropy robust Mendelian randomization (MR) methods and causal mediation analysis. We described it on the “Title page” (Page 1). |
|  | INTRODUCTION |  |  |
| 2 | Background | Explain the scientific background and rationale for the reported study. What is the exposure? Is a potential causal relationship between exposure and outcome plausible? Justify why MR is a helpful method to address the study question | The scientific background and rationale are detailed as to why we consider this issue in the first and second paragraphs of the “Introduction” section (Pages 6-7).  This study adopts two approaches by considering two exposures with related DPP4 (DPP4 gene expression at the mRNA level and DPP4 inhibition) and then using MR to assess the causal mechanism, including causal relationship and biological mechanisms, between DPP4 and CVDs. The study design of how we consider these exposures (DPP4 DPP4 gene expression at the mRNA level and DPP4 inhibition) can be found in the subsection “Study Design and Participants” in the section Materials and Methods (Pages 7-8). These details are also described in “Figure S1. Study design for DPP4 gene expression at the mRNA level” and “Method S1. Study design for DPP4 inhibition” in Supplement 1.  In the subsection “Study Design and Participants” (Page 7) in the section Materials and Methods, we addressed three clinically pertinent queries framed in causal language. This study's schematic representation is provided to address how we used MR and mediation analysis to answer these causal questions (“Figure S1” (Page 3) and “eFigure S5.1 in Method S5” in Supplement 1). Furthermore, the introduction of weak IV and pleiotropy robust MR methods and mediation analysis are illuminated for promoting our study. (“Mendelian Randomization Analysis” and “Causal Mediation Analysis by MR approach” in Materials and Methods, Pages 11-12). |
| 3 | Objectives | State specific objectives clearly, including pre-specified causal hypotheses (if any). State that MR is a method that, under specific assumptions, intends to estimate causal effects | We specified our objective by causal language (Study Design and participants in the section Materials and Methods, Page 8). We explained what kind of weak IVs and pleiotropy robust MR methods we used to clarify causality and to elaborate on the related assumptions (Mendelian Randomization Analysis and Causal mediation analysis by MR approach Materials and Methods, Pages 10-12). |
|  | METHODS |  |  |
| 4 | Study design and data sources | Present key elements of the study design early in the article. Consider including a table listing sources of data for all phases of the study. For each data source contributing to the analysis, describe the following: |  |
|  | (a) | Setting: Describe the study design and the underlying population, if possible. Describe the setting, locations, and relevant dates, including periods of recruitment, exposure, follow-up, and data collection, when available. | For (a), (b), and (d): All essential information for data sources about GWAS and the definition of phenotyping we used in this study are described in these subsections: “Study Design and Participants” (Page 7) and “Phenotyping CVD and type-2 diabetes GWAS from the Individual Datasets from the UKB” (Page 9) in the Materials and Methods section. Details also can be found in “Method S2. Description of the eQTLGen, UK Biobank, FinnGen, and GTEx” (Pages 18-19), “Table S2. Information about the GWAS summary datasets” (Pages 41-42) and “Table S3. Definitions of disease phenotype for UK Biobank” (Page 43-44) in Supplement 1.  For (c): The criteria for controlling and selecting genetic variants are presented in "SNP selection for DPP4 gene expression at the mRNA level” (Pages 12-13) and “SNP selection for DPP4 inhibition” (Pages 13) in the Materials and Methods section. More details for selection of SNPs as IVs for DPP4 inhibition can be found in “Method S1. Study design for DPP4 inhibition” in Supplement 1. |
|  | (b) | Participants: Give the eligibility criteria, and the sources and methods of selection of participants. Report the sample size, and whether any power or sample size calculations were carried out prior to the main analysis |  |
|  | (c) | Describe measurement, quality control and selection of genetic variants |  |
|  | (d) | For each exposure, outcome, and other relevant variables, describe methods of assessment and diagnostic criteria for diseases |  |
|  | (e) | Provide details of ethics committee approval and participant informed consent, if relevant | The details of ethics committee approval are provided in the “Ethics approval section” (Pages 24-25). |
| 5 | Assumptions | Explicitly state the three core IV assumptions for the main analysis (relevance, independence and exclusion restriction) as well assumptions for any additional or sensitivity analysis | We explicitly stated the core IV assumptions of conventional MR and illustrated why we need novel MR in this study for all analyses in the subsection "Mendelian Randomization Analysis”, Materials and Methods section. Details are provided in Method S3-S5 in Supplement 1. |
| 6 | Statistical Methods: main analysis | Describe statistical methods and statistics used |  |
|  | (a) | Describe how quantitative variables were handled in the analyses (i.e., scale, units, model) | All necessary information about how genetic variants are used and how MR estimators (primary analysis: MR-RPAS; sensitivity analyses and additional analyses: GRAPPLE, BESIDE-MR, and debiased IVW) are performed can be found in subsection "Mendelian Randomization Analysis” in the Materials and Methods section. More details are provided in Method S3-S5 in Supplement 1. In addition, “eFigure S3.1 in Method S3” in Supplement 1 illustrates the overall structure of RAPS-based MR methods. Furthermore, for resolve the second query, MR-based mediation analysis was used (Page 11 in Main manuscript and Method S5 in Supplement 1). |
|  | (b) | Describe how genetic variants were handled in the analyses and, if applicable, how their weights were selected |  |
|  | (c) | Describe the MR estimator (e.g. two-stage least squares, Wald ratio) and related statistics. Detail the included covariates and, in case of two-sample MR, whether the same covariate set was used for adjustment in the two samples |  |
|  | (d) | Explain how missing data were addressed |  |
|  | (e) | If applicable, indicate how multiple testing was addressed |  |
| 7 | Assessment of assumptions | Describe any methods or prior knowledge used to assess the assumptions or justify their validity | We provided details about further test MR assumptions in "Assessment of MR assumptions" (Pages 13-14) within the Materials and Methods section. |
| 8 | Sensitivity analyses and additional analyses | Describe any sensitivity analyses or additional analyses performed (e.g. comparison of effect estimates from different approaches, independent replication, bias analytic techniques, validation of instruments, simulations) | We described the methods as sensitivity analyses or additional analyses (GRAPPLE, BESIDE-MR, and debiased IVW) that we performed in our study in the subsection "Mendelian Randomization Analysis” (Pages 9-11) in the Materials and Methods. More details can be found in Method S3-S5 in Supplement 1. |
| 9 | Software and pre-registration |  |  |
|  | (a) | Name statistical software and package(s), including version and settings used | The Software within the Methods section and "Data Availability" (Page 25) sections describe all statistical software and settings used. |
|  | (b) | State whether the study protocol and details were pre-registered (as well as when and where) |  |
|  | RESULTS |  |  |
| 10 | Descriptive data |  |  |
|  | (a) | Report the numbers of individuals at each stage of included studies and reasons for exclusion. Consider use of a flow diagram | Details are given in "Participant Characteristics" (Page 14) in the Results section. Moreover, Table S5 in Supplemental 1 described the baseline characteristics of the participants from the UKB. |
|  | (b) | Report summary statistics for phenotypic exposure(s), outcome(s), and other relevant variables (e.g. means, SDs, proportions) | We listed the detailed information of the summary statistics in CVD and type 2 diabetes GWAS from the “Individual Datasets from the UKB” (Page 9), “SNP selection for DPP4 gene expression at the mRNA level” (Pages 12-13), and “SNP selection for DPP4 inhibition” (Pages 13) within the Methods section. Furthermore, the information on the exposures-GWAS summary datasets used in multivariate MR is reported in eTable S8.1. in Method S8 in Supplemental 1. |
|  | (c) | If the data sources include meta-analyses of previous studies, provide the assessments of heterogeneity across these studies | The GRAPPLE mode-detection plot (Figure 1) and the posterior probability of inclusion (Table S12-S15 in Supplement 2) for each SNP in BESIDE-MR allow us to assess the heterogeneity of the effect of the genetic variants on the outcome across studies. |
|  | (d) | For two-sample MR:  i.  Provide justification of the similarity of the genetic variant-exposure associations between the exposure and outcome samples  ii.  Provide information on the number of individuals who overlap between the exposure and outcome studies | We provide this information in “Table S2. Information about the GWAS summary datasets” (Page 40) and “eTable S8.1. in Method S8” in Supplemental 1. |
| 11 | Main results |  |  |
|  | (a) | Report the associations between genetic variant and exposure, and between genetic variant and outcome, preferably on an interpretable scale | The criterion of the selection p-value and the number of SNPs in MR is given in **“**Table S4. The criterion of the selection p-value and the number of SNPs in MR” in Supplement 1. The associations between genetic variants and exposure for univariate MR (DPP4 on CVD) are in Table S1-S4 in Supplement 2. The details of selected SNPs for multivariate MR are in Table S10-S11 in Supplement 2.  All information about SNPs as IVs used in MR: DPP4 inhibition -> CVDs are described in Method S1 in Supplemental 1. Furthermore, Table S22-S25 in Supplemental 2 provided the details of selected SNPs for the mediation analysis: DPP4 gene expression at the mRNA level, type 2 diabetes, and various CVD. |
|  | (b) | Report MR estimates of the relationship between exposure and outcome, and the measures of uncertainty from the MR analysis, on an interpretable scale, such as odds ratio or relative risk per SD difference | MR estimates (MR-RAPS as the primary analysis) of the relationship between exposure and the outcome can be found in Figure 1 in the Main manuscript and “Method S6. The diagnostic plots for MR-RAPS” (Pages 52-53) are in Supplement 1. More detail about can be found in the causal effect of DPP4 gene expression at the mRNA level and DPP4 inhibition on all-cause HF and other CVD in the “Results” section as follows: “Identifying potential pleiotropic pathways for the effect of DPP4 gene expression at the mRNA level on CVD” (pages 14-15), “Assess the causality of DPP4 gene expression at the mRNA level on CVDs” (pages 15-16) and “Assess the causality of DPP4 inhibition on CVDs” (pages 16-17). |
|  | (c) | If relevant, consider translating estimates of relative risk into absolute risk for a meaningful time period | None |
|  | (d) | Consider plots to visualize results (e.g. forest plot, scatterplot of associations between genetic variants and outcome versus between genetic variants and exposure) | None |
| 12 | Assessment of assumptions |  |  |
|  | (a) | Report the assessment of the validity of the assumptions | The detailed information can be found in the “Assessment of MR assumptions” (Pages 13-14) in the Methods section, in which the validity of the MR assumptions was evaluated using MR-RAPS, with a specific focus on InSIDE, using diagnostic and quantile-quantile (Q-Q) plots (“Method S6” in Supplement 1). |
|  | (b) | Report any additional statistics (e.g., assessments of heterogeneity across genetic variants, such as *I^2^*, Q statistic or E-value) | For univariate MR: The GRAPPLE mode-detection plot (Figure 1) and the posterior probability of inclusion (Table S12-15 in Supplement 2) for each SNP in BESIDE-MR allow us to assess the heterogeneity of the effect of the genetic variants on the outcome across studies.  For multivariate MR: to assess the strength of the instruments by detecting heterogeneity, the modified conditional Cochran's Q statistic for multivariate MR estimation is considered (eTable S8.2.-S8.3 in “Method S8”, Supplement 1). |
| 13 | Sensitivity analyses and additional analyses |  |  |
|  | (a) | Report any sensitivity analyses to assess the robustness of the main results to violations of the assumptions | We used GRAPPLE and BESIDE-MR as sensitivity analyses, BESIDE-MR with penalization, and the debiased IVW as additional analyses. The debiased IVW estimator mitigates the winner's curse bias without requiring a third independent GWAS to select IVs. |
|  | (b) | Report results from other sensitivity analyses or additional analyses | Details are described in “Assessment of MR Assumptions” (Pages 13-14 in the Materials and Methods section and “Reliability” (Page 17) in the Results. Also, all MR results can be found in the Results section's causal effect of DPP4 gene expression at the mRNA level and DPP4 inhibition on all-cause HF and other CVD as follows subsections: “Identifying potential pleiotropic pathways for the effect of DPP4 gene expression at the mRNA level on CVD” (pages 14-15), “Assess the causality of DPP4 gene expression at the mRNA level on CVDs” (pages 14-15) and “Assess the causality of DPP4 inhibition on CVDs” (pages 16-17). |
|  | (c) | Report any assessment of direction of causal relationship (e.g., bidirectional MR) | None |
|  | (d) | When relevant, report and compare with estimates from non-MR analyses | None |
|  | (e) | Consider additional plots to visualize results (e.g., leave-one-out analyses) | None |
|  | DISCUSSION |  |  |
| 14 | Key results | Summarize key results with reference to study objectives | The first paragraph (Pagse 17-18) summarizes all results in the “Discussion” section. |
| 15 | Limitations | Discuss limitations of the study, taking into account the validity of the IV assumptions, other sources of potential bias, and imprecision. Discuss both direction and magnitude of any potential bias and any efforts to address them | The last paragraph (Pages 21-23) in the “Discussion” section reported the study's limitations. |
| 16 | Interpretation |  |  |
|  | (a) | Meaning: Give a cautious overall interpretation of results in the context of their limitations and in comparison with other studies | Paragraphs 2 to 4 (Pages 18-20) in the “Discussion” section are the overall interpretation of results and comparison with other studies. |
|  | (b) | Mechanism: Discuss underlying biological mechanisms that could drive a potential causal relationship between the investigated exposure and the outcome, and whether the gene-environment equivalence assumption is reasonable. Use causal language carefully, clarifying that IV estimates may provide causal effects only under certain assumptions | The fourth paragraph (Pages 19-20) in the “Discussion” section discussed underlying biological mechanisms due to the heterogeneous gene. |
|  | (c) | Clinical relevance: Discuss whether the results have clinical or public policy relevance, and to what extent they inform effect sizes of possible interventions | Paragraphs 2 to 5 (Pages 17-20) in the Discussion and Conclusions sections discussed and presented the clinical relevance and importance. |
| 17 | Generalizability | Discuss the generalizability of the study results (a) to other populations, (b) across other exposure periods/timings, and (c) across other levels of exposure | The last paragraph(Pages 21-23) in the “Discussion” section discusses the generalizability of the study results about other populations. |
|  | OTHER INFORMATION |  |  |
| 18 | Funding | Describe sources of funding and the role of funders in the present study and, if applicable, sources of funding for the databases and original study or studies on which the present study is based | We have reported all funding sources in the “Funding” (Pages 23-24) in the Acknowledgments. |
| 19 | Data and data sharing | Provide the data used to perform all analyses or report where and how the data can be accessed, and reference these sources in the article. Provide the statistical code needed to reproduce the results in the article, or report whether the code is publicly accessible and if so, where | We reported detailed information in the "Data availability" (Page 25) subsections. |
| 20 | Conflicts of Interest | All authors should declare all potential conflicts of interest | We reported that no competing interests were declared within the “Disclosures” section (Pages 24-25). |

**Table S2. Information about the GWAS summary datasets**

The columns contain the phenotypes that are the exposure or the outcome used in this study, the dataset name stands for the consortium we acquire data, the population and sample size are considered in our study, and the Uniform Resource Locator (URL) of the dataset is the website we used to apply or download the datasets.

| Roles | Phenotype | Dataset name | Population | Sample size | URL of Dataset |
| --- | --- | --- | --- | --- | --- |
| Exposure | DPP4 gene expression at the mRNA level | eQTLGen Consortium | primarily European descendants | 31,684 | <https://eqtlgen.org/index.html> |
| Exposure | DPP4 inhibition | GTEx Portal | European ancestry | nearly 1000 | <https://www.gtexportal.org/home/> |
| - | HbA1c | OpenGWAS project | European | 45,734 | <https://gwas.mrcieu.ac.uk/datasets/ieu-b-4842/> |
| Outcome | All-cause Heart Failure | UK Biobank | British | 443,107 | <https://www.ukbiobank.ac.uk/> |
| Outcome | Atrial Fibrillation | UK Biobank | British | 443,107 | <https://www.ukbiobank.ac.uk/> |
| Outcome | Myocardial Infarction | UK Biobank | British | 443,107 | <https://www.ukbiobank.ac.uk/> |
| Outcome | Stroke | UK Biobank | British | 443,107 | <https://www.ukbiobank.ac.uk/> |
| Mediator | Type 2 diabetes mellitus | UK Biobank | British | 443,107 | <https://www.ukbiobank.ac.uk/> |
| Mediator | Type 2 diabetes mellitus | FinnGen project R9 | European descendants | 377,277 | <https://r9.finngen.fi/pheno/E4_DM2NASCOMP> |

**Table S3. Definitions of disease phenotype for UK Biobank**

| **Disease** | **Dataset name** | **ICD-9** | **ICD-10** | **Self-report code** | **Other** |
| --- | --- | --- | --- | --- | --- |
| All-cause Heart Failure | UK Biobank | 4254, 4280, 4281, 4289 | I11.0, I13.0, I13.2, I25.5, I42.0, I42.5,I42.8, I42.9, I50.0, I50.1, I50.9 | 1076 | Excluded ICD-10: I42.1, I42.2; Excluded Self-report: 1588 |
| Atrial Fibrillation | UK Biobank | 4273 | I48 | 1471 | - |
| Myocardial Infarction | UK Biobank | 410-412 | I21-I23, I24.1, I25.2 | 1075 | - |
| Stroke | UK Biobank | 430,431, 434, 436 | I60-I64 | 1081 | - |
| Type 2 diabetes mellitus | UK Biobank | - | E11, E14 | - | - |

**Method S3.** **Introduction of MR-RAPS, GRAPPLE, and BESIDE-MR**

The conventional Mendelian randomization (MR) analysis for two-sample summary-based data, such as the inverse variance weight (IVW) estimator[7], median methods[8], and MR-Egger[9], usually require most IVs to be sufficiently strong to avoid a weak IV bias and an unrealistic assumption, the instrument strength independent of direct Effect (InSIDE) assumption[9, 10] which means the magnitude of the pleiotropy is independent of the SNP-exposure associations. It can be viewed as a weaker version of the exclusion restriction assumption. In this study, we considered robust and novel methods of two-sample MR analysis, such as Mendelian randomization using the robust adjusted profile score (MR-RAPS)[11], the framework Genome-wide mR Analysis under Pervasive PLEiotropy (GRAPPLE)[12], and Bayesian set identification Mendelian randomization (BESIDE-MR)[13]. These methods belong to RAPS-based MR and are more robust to weak IV bias and the pleiotropic effect than conventional MR and can be used to distinguish multiple biological pathways. eFigure S3.1 illustrates the overall structure of RAPS-based MR methods.

**eFigure S3.1 The structure of RAPS-based MR methods**


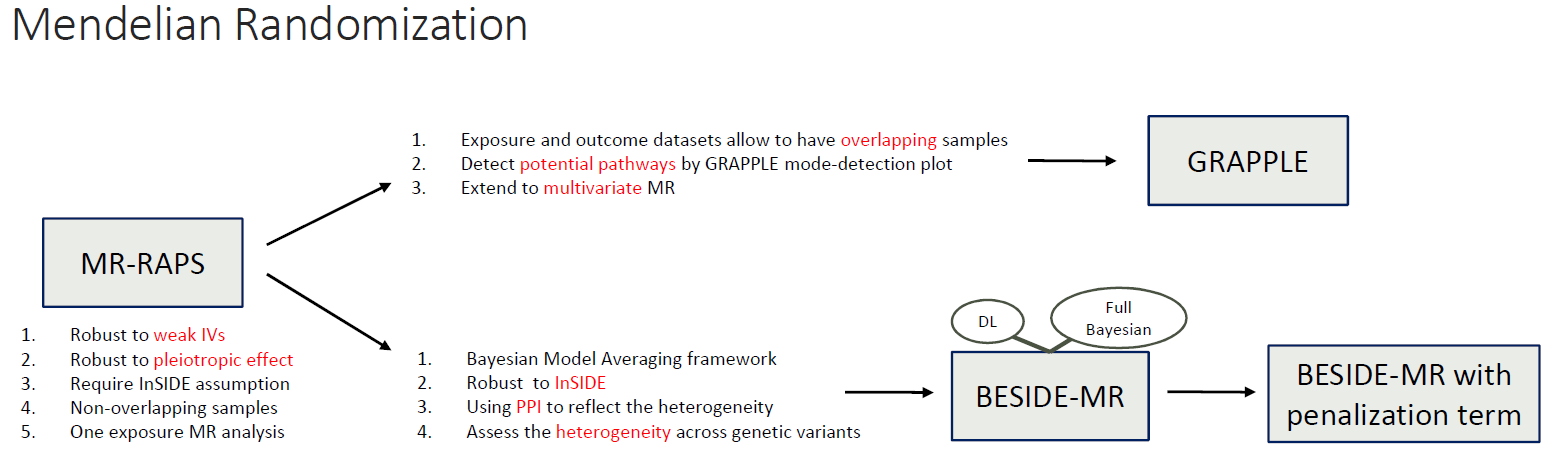


Using Tukey’s Biweight loss to robust outliers, MR-RAPS is a summary-based MR approach developed based on robust adjusted profile scores. It provides a consistent and asymptotically normal estimator. However, this method uses the profile likelihood of the summary data to estimate the causal effect, such that the causal estimate might not be globally identifiable and only the local identification is provable. GRAPPLE and BESIDE-MR are comprehensive frameworks based on MR-RAPS. We mainly identify potential mechanistic heterogeneity based on two MR methods: GRAPPLE and BESIDE-MR.

Like MR-RAPS, GRAPPLE is also a robust profile-likelihood approach that can efficiently use both strong and weak genetic instruments, but it allows the exposure-GWAS to have an overlapping sample with the GWAS outcome. This is not the case for MR-RPAS. GRAPPLE is also a visualization tool that can detect multiple pleiotropic pathways via the number of modes of the profile likelihood, domain knowledge, and previous research. Consider the robust profile likelihood in cases with no pleiotropy existing or the InSIDE assumption is satisfied; when a unique mode of profile likelihood occurs, the likelihood variable where the mode occurs is near the actual causal effect between the exposure and outcome. However, in cases with nonuniqueness of modes, the likelihood variable in which this mode occurs may represent the effect, which is similar to a combination of the actual causal effect between the exposure and outcome and the confounding effect between the exposure and outcome, induced by the heterogeneous genetic instruments related to a mode (for more discussion see Fig.1. Model overview and identification of pleiotropic pathways via multi-modality diagnosis in the Materials and Methods section in GRAPPLE [12]).

Moreover, the heterogeneity of the SNPs might influence the correlated horizontal pleiotropy and thus violate the exclusion restriction. Therefore, it was necessary to find and adjust these confounding risk factors to increase the strength of our causal estimates. Therefore, we conducted a multivariate MR analysis under the GRAPPLE framework. We adjusted for confounding risk factors to reduce correlated horizontal pleiotropy and evaluated how multiple pathways affected the causal effect of the exposure on the outcome. Therefore, we used the following workflow with GRAPPLE.[12] First, we identified multiple pleiotropic pathways via the modes of the profile likelihood in the GRAPPLE mode-detection plot. Then, we estimated the causal effect of the exposure on the outcome if a unique mode existed in the profile likelihood. Otherwise, we added possible confounding risk factors that could arise through the heterogeneity of SNPs and then performed multi-GRAPPLE to derive the correct causal estimate. The modified conditional Cochran's Q statistic for multi-MR was evaluated to assess the strength of IVs by detecting heterogeneity.[14, 15]

The one and two-component BESIDE-MR also relies on MR-RAPS to define the posterior probability distribution. In addition, it uses Bayesian Model Averaging (BMA), which exploits the Metropolis-Hastings (M-H) algorithm to search the parameter space of models with the highest posterior likelihood. There are two implementations of the M-H algorithm for estimating the pleiotropy variance: the full Bayesian approach and the DerSimonian-Laird (DL) approach, which uses iteration and the closed-form DerSimonian-Laird (DL) estimate[16] for estimating the pleiotropy variance, respectively. To explore the mechanism further, we used a two-component BESIDE-MR model, which allows a large proportion of SNPs to violate the InSIDE assumption, to classify the SNPs into multiple clusters where each cluster represented a different pathway by which the exposure affected the outcome. Moreover, for one component model, the posterior probability of inclusion (PPI) in the valid instrument set for each SNP is evaluated. SNP with large PPI is suggested to be classified into the valid instrument set. The SNP with PPI> 0.75 means it has strong evidence for inclusion in the cluster. This gives the reference for each SNP that should be classified to each cluster. Although the two-component model BESIDE-MR allows for a larger number of violating SNPs, each cluster must be evaluated using at least five instruments to avoid inaccurate estimation of pleiotropy variance[13].

Finally, the sensitivity for the one component BESIDE-MR, BESIDE-MR with penalization, is used to evaluate the average number of SNPs that should lie in the model. The positive penalization parameter will force the model to include more SNPs; however, the model with the negative parameter tends to contain fewer SNPs.[13] The actual causal effect for a binary outcome case was identified with a mean effect/beta up to the positive constant.[11] The causal odds ratio (OR) was derived from the exponential function of the mean effect/beta. The CI stands for confidence interval in frequentist statistics for MR-RAPS and GRAPPLE or credible interval in Bayesian statistics for BESIDE-MR.

**Method S4. Introduction of the debiased inverse-variance weighted estimator**

The winner's curse is one of the core issues in the MR study. To mitigate the winner's curse bias without requiring a third independent GWAS to select IVs. Ye T et al. posted a modification of the classical IVW estimator. The debiased inverse-variance weighted estimator (dIVW)[17] has addressed the theoretical properties of the IVW estimator, including their asymptotic properties under the classical MR setting with many weak instruments. In addition, it is more robust to many weak instruments and handles balanced horizontal pleiotropy than the classical IVW. The CI stands for confidence interval in frequentist statistics.

**Method S5. Causal mediation analysis by MR approach**

We considered the causal mediation model shown in eFigure S5.1 to explore the biological mechanisms among DPP4 gene expression at the mRNA level (exposure), type 2 diabetes (mediator), and each CVD (outcome). In this analysis, we used muti-GRAPPLE to implement causal mediation analysis[18, 19] to evaluate the direct effect of DPP4 gene expression at the mRNA level on each CVD, independent of type 2 diabetes mediation. The multi-MR estimates the effect of DPP4 gene expression on the risk of each CVD, adjusting for the genetic effect of type-2 diabetes. To calculate the direct effect of DPP4 gene expression on the risk of each CVD, we used the difference in coefficients method as our primary method. The direct effect (red arrow line in eFigure S5.1) was the estimate of DPP4 gene expression on the risk of each CVD with adjustment for type-2 diabetes. The direct effect was then subtracted from the total effect, estimated using MR, to estimate the indirect effect of the estimate of DPP4 gene expression on the risk of each CVD via type-2 diabetes (green arrow line in eFigure S5.1).

The results for type 2 diabetes using the UK Biobank data are presented in eFigure S5.2, while the results using FinnGen R9 data can be found in eFigure S5.3. Overall, for both two data sources for type 2 diabetes, we found that mediation analysis suggests sufficient evidence to support that DPP4 gene expression at the mRNA level directly affects HF, which type 2 diabetes does not mediate.

**eFigure S5.1** The causal mediation model

**
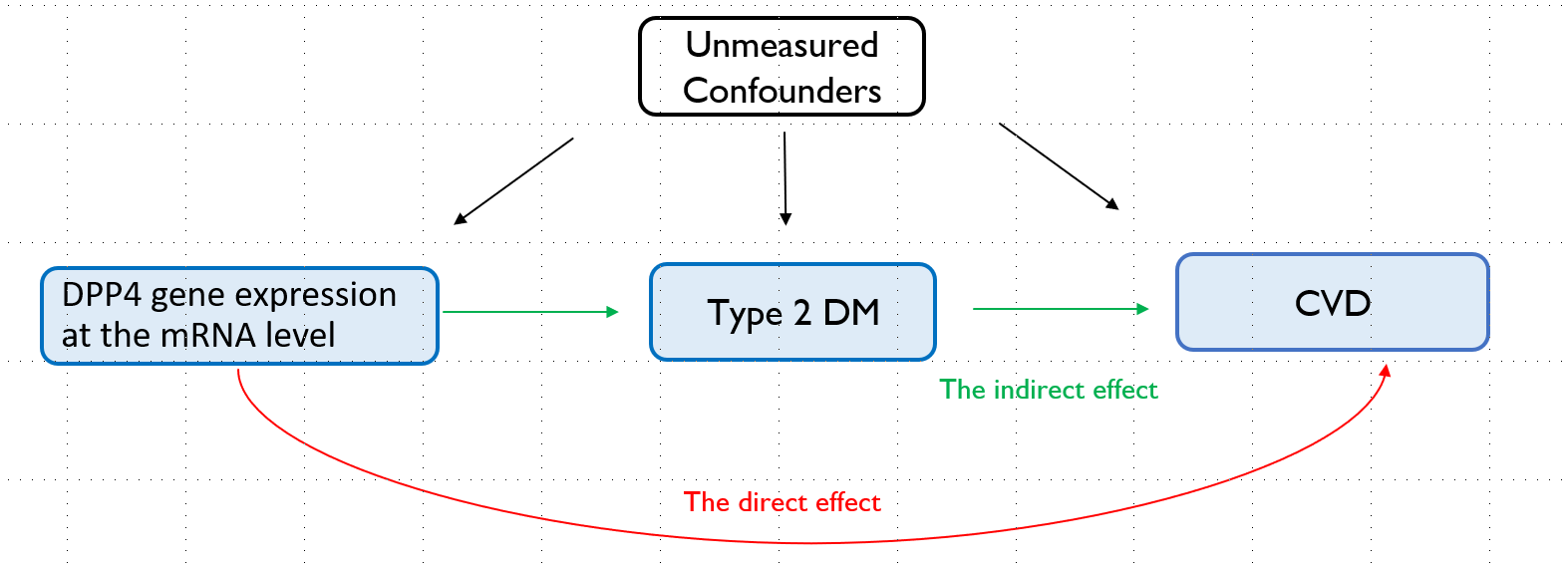
**

Exposure DPP4 gene expression at the mRNA level, mediator type 2 diabetes, each CVD (HF, AF, MI or stroke), and unmeasured confounder are considered in the causal mediation model. The red line stands for the direct effect of DPP4 gene expression at the mRNA level on CVD, and the green line stands for the indirect effect of DPP4 gene expression at the mRNA level on CVD.

**eFigure S5.2**
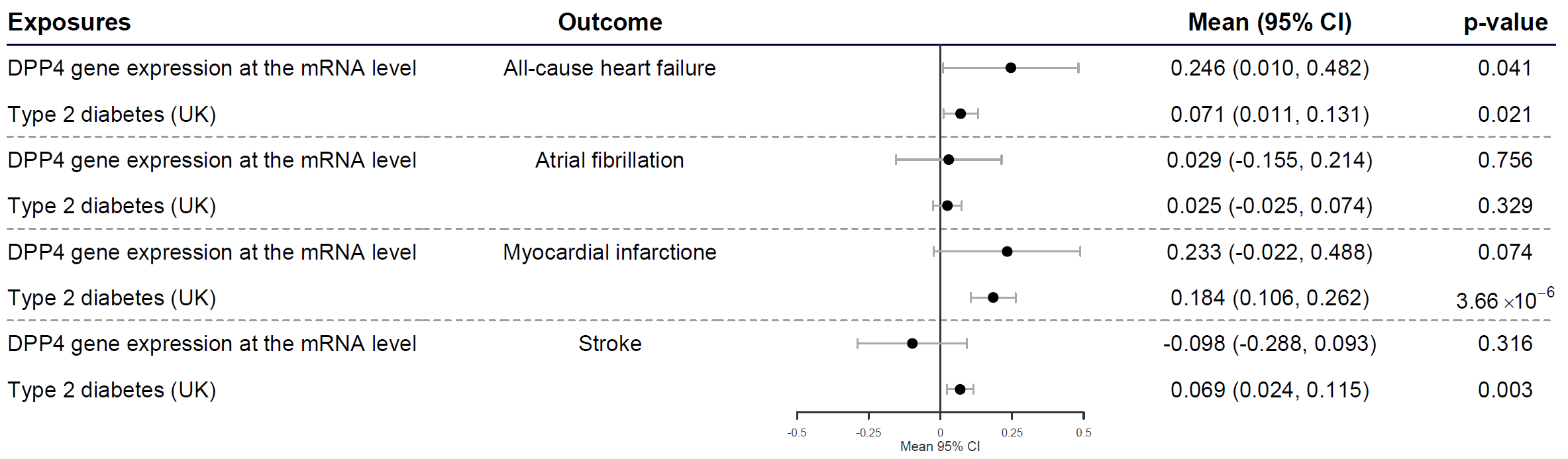


**eFigure S5.3**

**
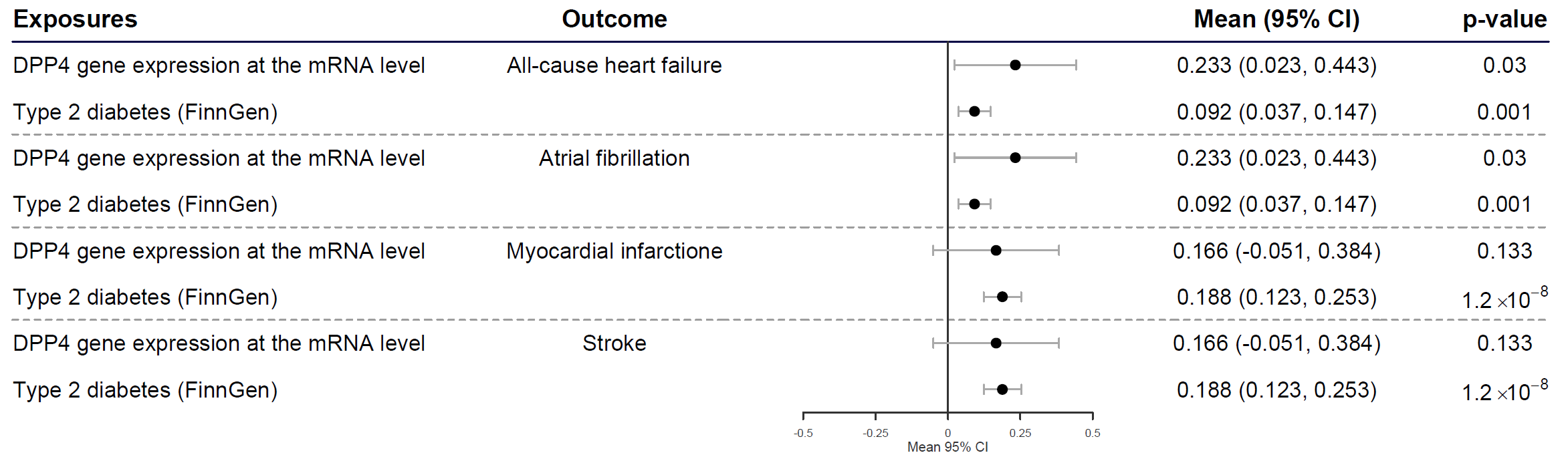
**

**Table S4.** **The criterion of the selection p-value and the number of SNPs in MR**

| **MR analysis for DPP4 gene expression at the mRNA level and CVDs** | **The criterion of the p-value** | **The number of SNPs** |
| --- | --- | --- |
| DPP4 gene expression at the mRNA level 🡪 All-cause heart failure | 1$\times$10^-4^ | 9 |
| DPP4 gene expression at the mRNA level 🡪 Atrial Fibrillation | 2$\times$10^-4^ | 9 |
| DPP4 gene expression at the mRNA level 🡪 Myocardial Infarction | 1$\times$10^-4^ | 9 |
| DPP4 gene expression at the mRNA level 🡪 Stroke | 1$\times$10^-4^ | 8 |

**Method S6.** **The diagnostic plots for MR-RAPS**

Although our preliminary analysis, MR-RAPS, was more robust than conventional MR methods, it needed to meet the InSIDE assumption.[11] Therefore, we used the diagnostic and Q-Q plots suggested in the Model Diagnostics section suggested by Zhao et al.[20] To evaluate the assumptions made in MR-RAPS, particularly whether the InSIDE assumption was violated. In addition, the heterogeneity p-values were computed by testing the null model in the linear regression of the standardized residual on the absolute weight. It was over 0.48 for HF, 0.76 for AF, 0.70 for MI, and 0.95 for stroke, indicating insufficient evidence to reject the InSIDE assumption; however, the InSIDE assumption does not mean it is true when a lack of sufficient evidence to falsify [20]. Here, we present the diagnostic plots showing the MR-RAPS for determining the causal effect of DPP4 gene expression at the mRNA level on CVD in eFigures S6.1-S6.4 below. For each figure, most standardized residuals (y-axis) should be approximately independent of the instrument weight (x-axis) investigated by the scatter plot in the left panel and roughly follow the standard normal distribution investigated by the Q-Q plots in the right panel.[20]

**eFigure S6.1. Diagnostic plots for DPP4 gene expression at the mRNA level on all-cause heart failure**


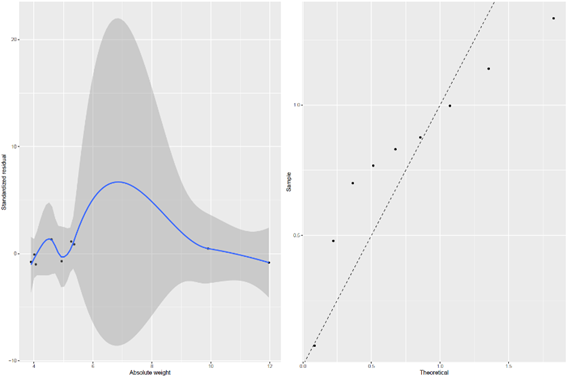


**eFigure S6.2. Diagnostic plots for DPP4 gene expression at the mRNA level on atrial fibrillation**


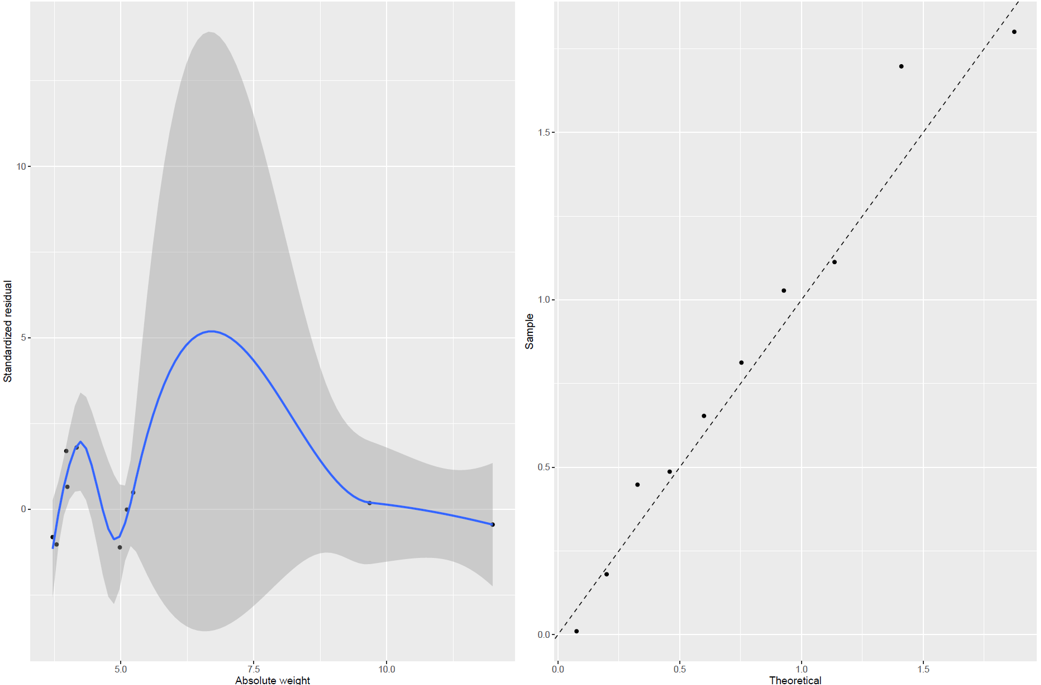


**eFigure S6.3. Diagnostic plots for DPP4 gene expression at the mRNA level on myocardial infarction**


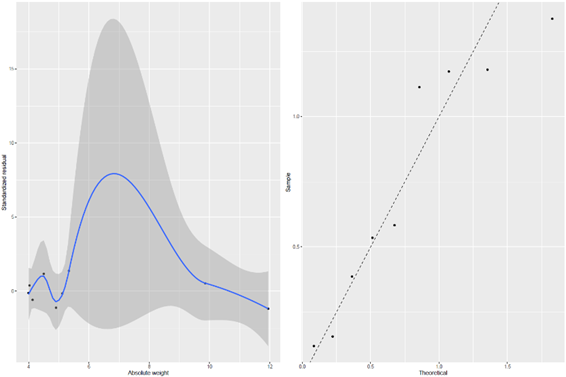


**eFigure S6.4. Diagnostic plots for DPP4 gene expression at the mRNA level on stroke**


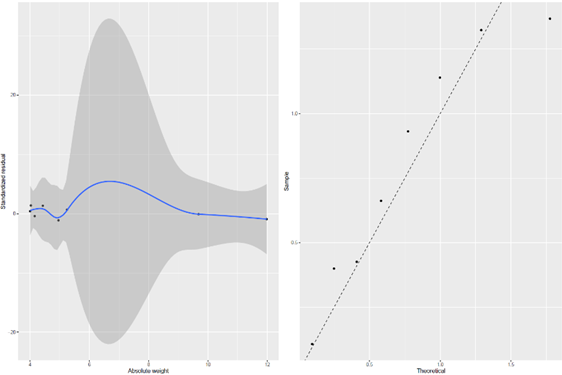


**Table S5. Baseline characteristics of the participants**

|  | **UK Biobank** | **FinnGen R9** |
| --- | --- | --- |
| **Participant (#N)** | 443,107 | 377,277 |
| **SNP (#N)** | 6,215,253 | 20,175,454 |
| **Age (Mean**$\boldsymbol{\pm}$**SD)** | 57.89 ± 8.35 | - |
| **Sex (Female %)** | 54.05% | 55.90% |
| **BMI (Mean**$\boldsymbol{\pm}$**SD; unit: kg/m^2^)** | 27.41 ± 4.78 | - |
| **All-cause heart failure** | | |
| Control | 428,229 | - |
| Case | 14,878 | - |
| **Atrial Fibrillation** | | |
| Control | 410,911 | - |
| Case | 32,196 | - |
| **Myocardial Infarction** | | |
| Control | 419,848 | - |
| Case | 23,259 | - |
| **Stroke** | | |
| Control | 427,341 |  |
| Case | 15,766 |  |
| **Type 2 Diabetes** | | |
| Control | 409,065 | 308,280 |
| Case | 34,042 | 46,373 |

**Method S7. Identified potential pleiotropic pathways**

This study mainly identifies potential mechanistic heterogeneity based on two powerful MR methods:

GRAPPLE workflow and BESIDE-MR. The GRAPPLE workflow was used to identify potential pleiotropic pathways for each disease induced by heterogeneous genetic instruments. From the GRAPPLE mode plots (Figure 1 in the main manuscript), a unique mode of the profile likelihood for the effect of DPP4 gene expression at the mRNA level on all-cause HF or AF was identified, indicating that no pleiotropic pathway exists from DPP4 gene expression at the mRNA level to all-cause HF or AF. A similar evaluation of other cardiovascular diseases (CVDs) showed three modes of profile likelihood for DPP4 gene expression at the mRNA level to myocardial infarction (MI) (mode1 at -0.1245; mode2 at 0.2635; mode3 at 1.0626) and two modes of profile likelihood for DPP4 gene expression at the mRNA level to stroke (mode1 at -0.2205; mode2 at 0.7346). The GRAPPLE analysis implied that three and two pleiotropic pathways exist for DPP4 gene expression at the mRNA level to MI and stroke, respectively. The evaluation of related genes, single nucleotide polymorphisms (SNPs), and diseases were conducted as detailed in Table S5-S9 in Supplement 2.

Regarding the pathway from DPP4 gene expression at the mRNA level to MI (Table S5-S7 in Supplement 2), the SNPs associated with mode1 were related to DPP4 gene expression at the mRNA level, those from mode2 were associated with BMI and SLC4A10, and those from mode3 were related to the total cholesterol (TC). In addition, the results from Table S8 and S9 in Supplement 2 indicate that mode1 SNPs in the pathway from DPP4 gene expression at the mRNA level to stroke are related to DPP4 gene expression at the mRNA level, and mode2 SNPs are linked to hypertension (HTN) and total triglyceride (TG) levels. Therefore, we found that BMI, SLC4A10, and TC were implicated in the potential pleiotropic pathways from SNPs associated with DPP4 gene expression at the mRNA level to MI. HTN and TG appear to play similar roles in pathways from SNPs associated with DPP4 gene expression at the mRNA level to stroke.

The one-component BESIDE-MR produced similar results to the preceding MR for the pathway from DPP4 gene expression at the mRNA level to MI and stroke. We also estimated the pleiotropy variance (eTable S7.1 in Supplement 1) and PPI for the valid instrument set for each SNP (Table S12-S15 in Supplement 2 ) for each case. Using the criterion PPI>0.75, we found that around 80% of SNPs lie in the valid instrument set for cases HF and AF, while only around 50% of SNPs did so for MI and stroke, in which SNPs rs635634 on ABO gene and rs34372695 on RAB25 gene are often classified as invalid. Also, rs12619850 on SLC4A10 and rs635634 on ABO related to TC lie in the invalid set by BESIDE-MR for MI, which is the role of potential risk factors suggested by GRAPPLE. Therefore, BESIDE-MR provides another reasonable motivation for adjusting SLC4A10 and TC.

**eTable S7.1 Estimation of the effect (beta) and pleiotropy variance by BESIDE-MR**

| **Outcome** | **Parameters*** | **Estimator** | **Mean** | **Credible Intervals (CI)** |
| --- | --- | --- | --- | --- |
| All-cause heart failure | $\beta$ | DL estimate | 0.13 | (-0.08, 0.33) |
|  |  | Full Bayesian | 0.32 | (0.07, 0.62) |
|  | $\tau^{2}\times{10}^{4}$ | DL estimate | 1.75 | 0 |
|  |  | Full Bayesian | 0.64 | (0.09, 3.04) |
| Atrial fibrillation | $\beta$ | DL estimate | -0.01 | (-0.13, 0.11) |
|  |  | Full Bayesian | 0.01 | (-0.12, 0.15) |
|  | $\tau^{2}\times{10}^{4}$ | DL estimate | 0.01 | 0 |
|  |  | Full Bayesian | 0.29 | (0.08, 0.96) |
| Myocardial infarction | $\beta$ | DL estimate | -0.01 | (-0.28, 0.42) |
|  |  | Full Bayesian | 0.09 | (-0.23, 0.59) |
|  | $\tau^{2}\times{10}^{4}$ | DL estimate | 1.44 | 0 |
|  |  | Full Bayesian | 1.09 | (0.09, 6.67) |
| Stroke | $\beta$ | DL estimate | 0.01 | (-1.40, 1.60) |
|  |  | Full Bayesian | 0.13 | (-0.28, 0.80) |
|  | $\tau^{2}\times{10}^{4}$ | DL estimate | 1433.41 | (564.46, 3586.26) |
|  |  | Full Bayesian | 1.10 | (0.09, 6.84) |

We used 600000 iterations and 100000 burn-ins with the DL and fully Bayesian implementations for convergence. Parameters* $\beta:$ the causal effect parameter, $\tau^{2}:$ the pleiotropy variance. Mean stands for the mean of the posterior of the effect estimate.

**Figure S3. The odds ratio of the** **DPP4 gene expression at the mRNA level on CVD via** **univariate MR**
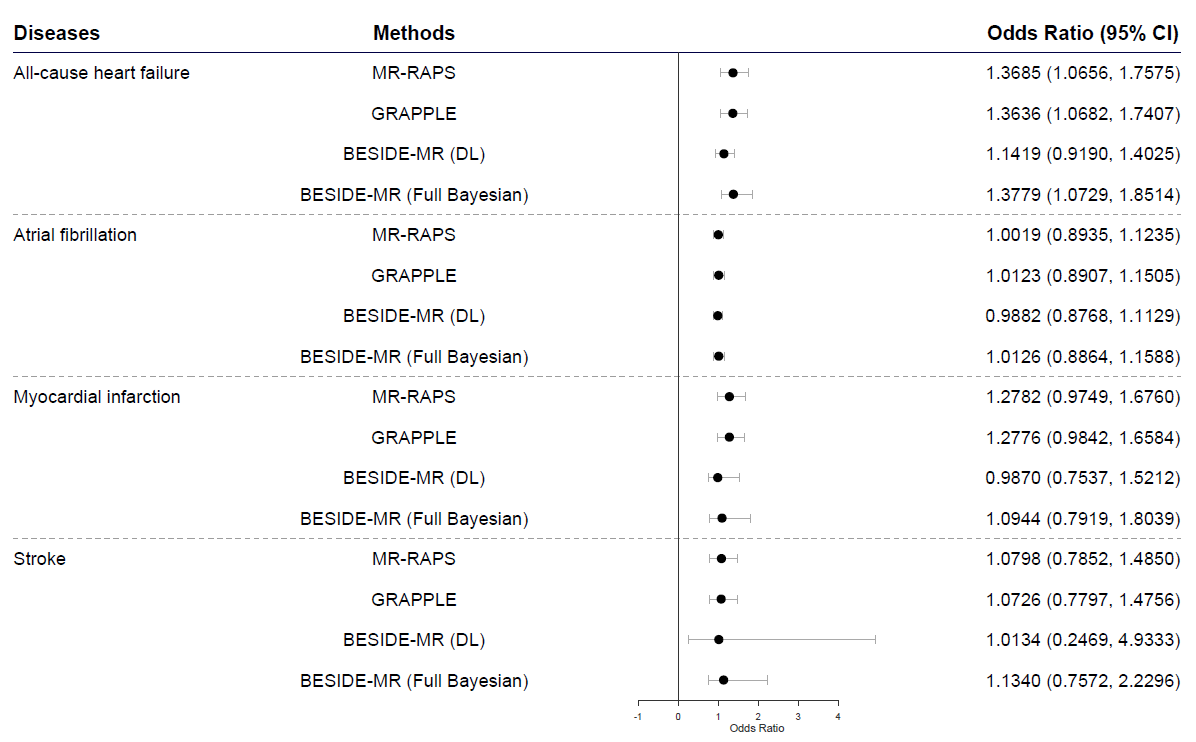
Results of univariate MR including estimated odds ratio (95% CI) per standard deviation increase of DPP4 gene expression at the mRNA level on CAD. Abbreviation: CI: confidence intervals for MR-GENIUS, MR-RAPS, and GRAPPLE; credible interval for BESIDE-MR. Mean: the mean causal effect; p-value: p-value of the mean causal effect.

**Method S8. The multivariate GRAPPLE for the pathway from DPP4 gene expression at the mRNA level to MI and stroke**

Continuing discussion in Method S7, the existence of multiple modes of the profile likelihood for the pathway from DPP4 gene expression at the mRNA level to MI or stroke prompted us to conduct multivariate MR analysis within the GRAPPLE framework. This approach enabled us to reduce the correlated horizontal pleiotropy and to obtain a more precise causal estimate by adjusting the selected candidates (SLC4A10, BMI, and TC for MI; HTN and TG for stroke) identified in the Method S7 in Supplemental 1. By following the GRAPPLE flow, we consider two multivariate GRAPPLE analyses, including that assessing the effects of DPP4 gene expression at the mRNA level, SLC4A10, BMI, and total cholesterol (TC) on MI and that assessing the effects of DPP4 gene expression at the mRNA level, hypertension (HTN), and total triglyceride (TG) on stroke, are given as follows. In addition, to assess the strength of the instruments by detecting heterogeneity, the modified conditional Cochran's Q statistic for multivariate MR estimation in the methodology articles [14, 21] is considered. Here, we follow the argument of this methodology article: Under the null hypothesis, the selected SNPs, as IV, do not contain enough information to predict all exposures. If all conditional Cochran's Q statistics for each exposure are larger than the chosen critical value of $\chi^{2}$-distribution, the null hypothesis of under-identification should be rejected, and the test suggested that these SNPs can predict variation in all exposures. [14] Consequently, we will show that insufficient evidence supports causality between DPP4 gene expression at the mRNA level and MI and stroke.

**Information about the GWAS summary datasets of exposures**

First, we describe the exposure-GWAS summary datasets used in the multivariate GRAPPLE in eTable S8.1. These studies, most of which used the GWAS VCF format, are from the website of the MRC Integrative Epidemiology Unit (IEU) OpenGWAS project (<https://gwas.mrcieu.ac.uk/> ).[22]

**eTable S8.1. The information on the exposures-GWAS summary datasets**

| **Phenotype** | **Dataset name** | **Citation** | **Population** | **Sample size** | **URL of Dataset** |
| --- | --- | --- | --- | --- | --- |
| DPP4 gene expression at the mRNA level | eQTLGen Consortium | Võsa, Urmo, et al.[4] | European | 31,684 | <https://eqtlgen.org/index.html> |
| SLC4A10 | eQTLGen Consortium | Võsa, Urmo, et al.[4] | European | 31,684 | <https://eqtlgen.org/index.html> |
| BMI | ieu-a-974 | Locke, Adam E., et al.[23] | European | 171,977 | <https://gwas.mrcieu.ac.uk/datasets/ieu-a-974/> |
| Total cholesterol | met-c-933 | Kettunen, Johannes, et al.[24] | European | 21,491 | <https://gwas.mrcieu.ac.uk/datasets/met-c-933/> |
| Hypertension | ebi-a-GCST008036 | Wojcik, Genevieve L., et al. [25] | Hispanic  or Latino | 21,936 | <https://gwas.mrcieu.ac.uk/datasets/ebi-a-GCST008036/> |
| Total triglyceride | ebi-a-GCST000758 | Teslovich, Tanya M., et al [26] | European | 96,598 | <https://gwas.mrcieu.ac.uk/datasets/ebi-a-GCST000758/> |

**The joint causal effect of DPP4 gene expression at the mRNA level, SLC4A10, BMI, and TC on MI**

We used the multivariate GRAPPLE method with a two-sample design to investigate the causal effects of DPP4 gene expression at the mRNA level, SLC4A10, BMI, and total cholesterol on myocardial infarction. The summary statistics sources were the eQTLGen Consortium, Locke et al.[23], and Kettunen et al.[24], respectively. The selected p-value of the multivariate GRAPPLE method was 1×10^-4^. We extracted only SNPs with a weak LD at a correlation coefficient square R^2^ < 0·001. The number of SNPs was 49. The detailed SNPs as IVs are shown in Table S10 in Supplement 2. The results of the multivariate GRAPPLE analysis on the pathway from DPP4 gene expression at the mRNA level, SLC4A10, BMI, and TC to MI (eFigure S8.1) indicated that increasing the level of TC could increase the risk of MI with a causal OR of 1.63 [95% CI, 1.43–1.88] and an effect/beta of 0.49(95% CI, 0.36–0.63; p = 1.37 × 10-12). Furthermore, DPP4 gene expression at the mRNA level, SLC4A10, and BMI did not significantly affect the risk of MI, further suggesting that SLC4A10 and BMI may not be a mediator of the effect of DPP4 gene expression at the mRNA level on MI risk.

Moreover, the modified conditional Cochran's Q-statistics, degrees of freedom, and the corresponding p-values are reported in eTable S8.2. The critical value for $\chi^{2}$-distribution with degrees of freedom 45 at the 5% level is 61.7, which is not larger than Cochran's Q-statistics for each exposure (DPP4 gene expression at the mRNA level, SLC4A10, BMI, and TC). Thus, the null hypothesis of under-identification can be rejected, and SNPs can predict variation in these exposures suggested by the test.

**eFigure S8.1. The joint causal effect of multivariate MR with muti-exposures that including DPP4 gene expression at the mRNA level, SLC4A10, BMI, and TC on the outcome MI
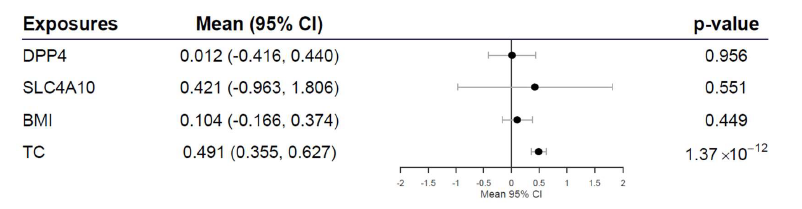
**The mean effects, 95% confidence intervals, and p-values are shown. Abbreviation: CI: confidence interval. Mean: the mean causal effect; p-value: p-value of the mean causal effect.

**eTable S8.2 The modified conditional Cochran's Q-statistics (p-values) of the multi-MR for DPP4 gene expression at the mRNA level on MI with adjusted for SLC4A10, BMI, and TC.**

| **Exposure** | **DPP4 gene expression at the mRNA level** | **SLC4A10** | **BMI** | **TC** |
| --- | --- | --- | --- | --- |
| Q-stat. (p-value) | 308.1  (1.18$\times{10}^{-39}$) | 78.0  (3.98$\times{10}^{-3}$) | 603.0  (5.04$\times{10}^{-97}$) | 414.8  (7.27$\times{10}^{-60}$) |
| df | 45 | | | |

Q-stat.: Modified conditional Cochran's Q-statistics. df: degrees of freedom (the df for Q-statistics be the number of instruments minus the number of exposures).

**The joint causal effect of DPP4 gene expression at the mRNA level, HTN, and total triglyceride on stroke**

We investigated the causal effect of DPP4 gene expression at the mRNA level, hypertension, and total TG on stroke. The summary statistics sources were the eQTLGen Consortium, Wojcik et al.[25], and Teslovich et al.[26], respectively. The selected p-value of the multivariate GRAPPLE method was 1×10^-4^. We extracted only SNPs with a weak LD at a correlation coefficient square R^2^ < 0·001. The number of SNPs was 56. The detailed SNPs as IVs are shown in Table S11, Supplement 2. The results of the multivariate GRAPPLE analysis for the pathway from DPP4 gene expression at the mRNA level, HTN, and TG to stroke (eFigure S8.2) showed that individuals with a diagnosis of hypertension had an elevated risk of stroke, with a causal OR of 1.27 [95% CI, 1.09–1.46] (effect: 0.24; 95% CI, 0.09–0.38; p = 0.0014). However, No significant effect was observed for DPP4 gene expression at the mRNA level and TG. (the lower part B of Figure 3). This suggests that we have no evidence that TG can mediate the effect of DPP4 gene expression at the mRNA level on stroke risk. All results indicate that the null hypothesis of under-identification should be rejected, and thus, the test suggested that these SNPs can predict variation in all exposures. In addition, the modified conditional Cochran's Q- Q-statistics, degrees of freedom, and the corresponding p-values are reported in eTable S8.3. The critical value for $\chi^{2}$-distribution with degrees of freedom 54 at the 5% level is 70.99, which is not larger than Cochran's Q-statistics for each exposure (DPP4 gene expression at the mRNA level, HTN, and TG). Therefore, the null hypothesis of under-identification can be rejected, and SNPs can predict variation in these exposures suggested by the test.

**eFigure S8.2 The joint causal effect of multivariate MR for DPP4 gene expression at the mRNA level, SLC4A10, BMI, and TC on MI**


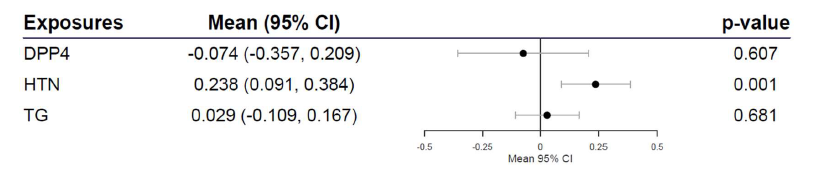


Q-stat.: Modified conditional Cochran's Q-statistics. df: degrees of freedom (the df for Q-statistics be the number of instruments minus the number of exposures).

**eTable S8.3. The modified conditional Cochran's Q-statistics (p-values) for the multi-MR for DPP4 gene expression at the mRNA level on stroke was adjusted for HTN and TG.**

| **Exposure** | **DPP4 gene expression at the mRNA level** | **HTN** | **TG** |
| --- | --- | --- | --- |
| **Q-stat.**  **(p-value)** | 314.1  (5.64$\times{10}^{-38}$) | 812.2  (2.94$\times{10}^{-135}$) | 2609.8  (0) |
| **df** | 53 | | |

Q-stat.: Modified conditional Cochran's Q-statistics. df: degrees of freedom.

**References**

1. Walker VM, Kehoe PG, Martin RM, Davies NM. Repurposing antihypertensive drugs for the prevention of Alzheimer’s disease: a Mendelian randomization study. *International Journal of Epidemiology* 2019; **49**:1132-1140.

2. Xu M, Zheng J, Hou T, Lin H, Wang T, Wang S*, et al.* SGLT2 inhibition, choline metabolites, and cardiometabolic diseases: a mediation Mendelian randomization study. *Diabetes Care* 2022; **45**:2718-2728.

3. Consortium G. The GTEx Consortium atlas of genetic regulatory effects across human tissues. *Science* 2020; **369**:1318-1330.

4. Võsa U, Claringbould A, Westra HJ, Bonder MJ, Deelen P, Zeng B*, et al.* Large-scale cis- and trans-eQTL analyses identify thousands of genetic loci and polygenic scores that regulate blood gene expression. *Nat Genet* 2021; **53**:1300-1310.

5. Kurki MI, Karjalainen J, Palta P, Sipilä TP, Kristiansson K, Donner KM*, et al.* FinnGen provides genetic insights from a well-phenotyped isolated population. *Nature* 2023; **613**:508-518.

6. Skrivankova VW, Richmond RC, Woolf BA, Yarmolinsky J, Davies NM, Swanson SA*, et al.* Strengthening the reporting of observational studies in epidemiology using Mendelian randomization: the STROBE-MR statement. *Jama* 2021; **326**:1614-1621.

7. Burgess S, Butterworth A, Thompson SG. Mendelian randomization analysis with multiple genetic variants using summarized data. *Genet Epidemiol* 2013; **37**:658-665.

8. Bowden J, Davey Smith G, Haycock PC, Burgess S. Consistent Estimation in Mendelian Randomization with Some Invalid Instruments Using a Weighted Median Estimator. *Genet Epidemiol* 2016; **40**:304-314.

9. Bowden J, Davey Smith G, Burgess S. Mendelian randomization with invalid instruments: effect estimation and bias detection through Egger regression. *Int J Epidemiol* 2015; **44**:512-525.

10. Bowden J, Del Greco M F, Minelli C, Davey Smith G, Sheehan N, Thompson J. A framework for the investigation of pleiotropy in two‐sample summary data Mendelian randomization. *Statistics in medicine* 2017; **36**:1783-1802.

11. Zhao Q, Wang J, Hemani G, Bowden J, Small DS. Statistical inference in two-sample summary-data Mendelian randomization using robust adjusted profile score. *The Annals of Statistics* 2020; **48**:1742-1769.

12. Wang J, Zhao Q, Bowden J, Hemani G, Davey Smith G, Small DS*, et al.* Causal inference for heritable phenotypic risk factors using heterogeneous genetic instruments. *PLoS Genet* 2021; **17**:e1009575.

13. Shapland CY, Zhao Q, Bowden J. Profile-likelihood Bayesian model averaging for two-sample summary data Mendelian randomization in the presence of horizontal pleiotropy. *Stat Med* 2022; **41**:1100-1119.

14. Sanderson E, Davey Smith G, Windmeijer F, Bowden J. An examination of multivariable Mendelian randomization in the single-sample and two-sample summary data settings. *International journal of epidemiology* 2019; **48**:713-727.

15. Sanderson E, Spiller W, Bowden J. Testing and correcting for weak and pleiotropic instruments in two‐sample multivariable Mendelian randomization. *Statistics in medicine* 2021; **40**:5434-5452.

16. DerSimonian R, Laird N. Meta-analysis in clinical trials. *Controlled clinical trials* 1986; **7**:177-188.

17. Ye T, Shao J, Kang H. Debiased inverse-variance weighted estimator in two-sample summary-data Mendelian randomization. *The Annals of statistics* 2021; **49**:2079-2100.

18. VanderWeele TJ. Mediation analysis: a practitioner's guide. *Annual review of public health* 2016; **37**:17-32.

19. Carter AR, Sanderson E, Hammerton G, Richmond RC, Davey Smith G, Heron J*, et al.* Mendelian randomisation for mediation analysis: current methods and challenges for implementation. *European journal of epidemiology* 2021; **36**:465-478.

20. Zhao Q, Chen Y, Wang J, Small DS. Powerful three-sample genome-wide design and robust statistical inference in summary-data Mendelian randomization. *International journal of epidemiology* 2019; **48**:1478-1492.

21. Zhao Q, Wang J, Miao Z, Zhang NR, Hennessy S, Small DS*, et al.* A Mendelian randomization study of the role of lipoprotein subfractions in coronary artery disease. *Elife* 2021; **10**:e58361.

22. Lyon MS, Andrews SJ, Elsworth B, Gaunt TR, Hemani G, Marcora E. The variant call format provides efficient and robust storage of GWAS summary statistics. *Genome biology* 2021; **22**:1-10.

23. Locke AE, Kahali B, Berndt SI, Justice AE, Pers TH, Day FR*, et al.* Genetic studies of body mass index yield new insights for obesity biology. *Nature* 2015; **518**:197-206.

24. Kettunen J, Demirkan A, Würtz P, Draisma HH, Haller T, Rawal R*, et al.* Genome-wide study for circulating metabolites identifies 62 loci and reveals novel systemic effects of LPA. *Nature communications* 2016; **7**:1-9.

25. Wojcik GL, Graff M, Nishimura KK, Tao R, Haessler J, Gignoux CR*, et al.* Genetic analyses of diverse populations improves discovery for complex traits. *Nature* 2019; **570**:514-518.

26. Teslovich TM, Musunuru K, Smith AV, Edmondson AC, Stylianou IM, Koseki M*, et al.* Biological, clinical and population relevance of 95 loci for blood lipids. *Nature* 2010; **466**:707-713.
